# Decoding the Regulatory Landscape of Ageing in Musculoskeletal Engineered Tissues Using Genome-Wide DNA Methylation and RNASeq

**DOI:** 10.1371/journal.pone.0160517

**Published:** 2016-08-17

**Authors:** Mandy Jayne Peffers, Katarzyna Goljanek-Whysall, John Collins, Yongxiang Fang, Michael Rushton, John Loughlin, Carole Proctor, Peter David Clegg

**Affiliations:** 1 Institute of Ageing and Chronic Disease, University of Liverpool, Leahurst, Chester High Road, Neston, Wirral, UK, CH64 7TE; 2 Thurston Arthritis Research Centre, School Of Medicine, University of North Carolina at Chapel Hill, Chapel Hill, North Carolina, USA, 27599; 3 Centre for Genomic Research, Institute of Integrative Biology, Biosciences Building, Crown Street, University of Liverpool, Liverpool, UK, L69 7ZB; 4 Musculoskeletal Research Group, Institute of Cellular Medicine, Newcastle University, Newcastle upon Tyne, UK, NE2 4HH; 5 Newcastle University Institute for Ageing, Newcastle University, Campus for Ageing and Vitality, Newcastle upon Tyne, UK, NE4 5PL; The University of Adelaide, AUSTRALIA

## Abstract

Mesenchymal stem cells (MSC) are capable of multipotent differentiation into connective tissues and as such are an attractive source for autologous cell-based regenerative medicine and tissue engineering. Epigenetic mechanisms, like DNA methylation, contribute to the changes in gene expression in ageing. However there was a lack of sufficient knowledge of the role that differential methylation plays during chondrogenic, osteogenic and tenogenic differentiation from ageing MSCs. This study undertook genome level determination of the effects of DNA methylation on expression in engineered tissues from chronologically aged MSCs. We compiled unique DNA methylation signatures from chondrogenic, osteogenic, and tenogenic engineered tissues derived from young; n = 4 (21.8 years ± 2.4 SD) and old; n = 4 (65.5 years±8.3SD) human MSCs donors using the Illumina HumanMethylation 450 Beadchip arrays and compared these to gene expression by RNA sequencing. Unique and common signatures of global DNA methylation were identified. There were 201, 67 and 32 chondrogenic, osteogenic and tenogenic age-related DE protein-coding genes respectively. Findings inferred the nature of the transcript networks was predominantly for ‘cell death and survival’, ‘cell morphology’, and ‘cell growth and proliferation’. Further studies are required to validate if this gene expression effect translates to cell events. Alternative splicing (AS) was dysregulated in ageing with 119, 21 and 9 differential splicing events identified in chondrogenic, osteogenic and tenogenic respectively, and enrichment in genes associated principally with metabolic processes. Gene ontology analysis of differentially methylated loci indicated age-related enrichment for all engineered tissue types in ‘skeletal system morphogenesis’, ‘regulation of cell proliferation’ and ‘regulation of transcription’ suggesting that dynamic epigenetic modifications may occur in genes associated with shared and distinct pathways dependent upon engineered tissue type. An altered phenotype in engineered tissues was observed with ageing at numerous levels. These changes represent novel insights into the ageing process, with implications for stem cell therapies in older patients. In addition we have identified a number of tissue-dependant pathways, which warrant further studies.

## Introduction

The limited ability of articular cartilage, bone and tendon to regenerate has prompted the development of cell-based tissue engineering techniques. One cell therapy option is mesenchymal stem cells (MSC); a heterogeneous population of multi-potent cells with the ability to differentiation into tissues including cartilage, bone and tendon, thus accommodating tissue repair and homeostasis. The principles of tissue engineering involve a multifarious interaction of factors, and knowledge of the extent MSC phenotype and differentiation capacity alter with ageing is limited. Subsequently, any loss in functionality with age would have profound consequences for the maintenance of tissue viability and the quality of tissues. MSCs have been utilised in clinical trials of cell therapies for cartilage repair and osteoarthritis (reviewed [1]), bone fracture treatment [2] and in a limited number of tendon therapies [3]. However, the therapeutic efficiency of MSCs for clinical applications remains limited, possibly due to the attenuation of their regenerative potential in aged patients with chronic diseases.

Advancing age is a prominent risk factor that is closely linked with the onset and progression of diseases such as osteoarthritis, osteoporosis and tendinopathy. Understanding the influence that ageing has on chondrogenic, osteogenic and tenogenic progenitor cells such as MSCs is important in determining how these processes affect their capacity to differentiate into functional chondrocytes, osteoblasts and tenocytes for use in therapeutic applications. A model using MSCs derived from young and old donors to musculoskeletal engineered tissues could aid in our understanding of musculoskeletal ageing.

To understand the underlying mechanisms that are responsible for age-related changes in musculoskeletal engineered tissues, a number of studies have been undertaken on ageing MSCs (reviewed [4]), as well as the differentiation potential of tissue engineered cartilage [5] and bone [6], though no studies have addressed these questions in tendon.

There are a few studies investigating the effect of chronological age of donor MSC on engineered tissues, some with conflicting findings. One study found a reduction in glycosaminoglycans in chondrogenesis with age [7] whereas another experiment using a wider donor age-range found no change [8]. Contrasting results of the chondrogenic differentiation potential of adult MSCs has been described with one study reporting age independence [9], whilst another demonstrated a negative correlation with advancing age in male but not female donors [10]. A study demonstrated that foetal and adult MSCs are differentially regulated by transforming growth factor-β stimuli to activate the onset of chondrogenesis, suggesting that discrete age-related mechanisms direct chondrogenic regulation following development and postnatal maturation [11].

Age-related changes in osteogenesis have been more widely studied. Osteogenic progenitors in MSCs derived from rat bone marrow demonstrated an age-related decline. In addition MSCs from young rats had a significantly greater bone formation capability *in vivo* compared with aged rats [12]. The osteogenic potential of MSCs is independent of advancing age in adult human donors [13]. However, a decline in the osteogenic precursor population, due to accelerated senescence and lower rate of population doublings in MSCs isolated from older donors suggests a reduction in osteoblast formation. This may contribute to the age-related reduction in bone formation in the elderly [14]. In age-related studies of osteogenic differentiation one group identified an increase in alkaline phosphatase with age [15] whilst another demonstrated a decrease [16]. It is thought these discrepancies could be due to the heterogeneous population which is propagated within and amongst donor populations.

Few studies have investigated the effects of ageing MSCs on tendon tissue engineering. However, one study on human tendon stem cells from aged tendons described reduced proliferation capacity and premature entry into senescence [17]. A recent study in rat tendon-derived stem cells from older donors demonstrated earlier entry into senescence which was postulated to be due to a reduction in the levels of miRNA-135a, a ROCK-1 targeting microRNA (miR) that blocks entry into senescence pathways. This may be due to a reduction in miR-135a, which binds to ROCK-1 and inhibits entry into senescence in young tendon. Thus a decrease in miR-135a in older tendon may be the cause of reduced stem cell proliferation, self-renewal and tenogenic differentiation [18].

The advent of global DNA methylation arrays and RNASeq studies have made it possible to explore gene methylation and/or expression during cell development [19], tissue differentiation [20], disease [21] and ageing [22, 23]. In addition, the global relationship between gene methylation and expression can now be investigated in ageing [24]. Whilst global methylation and RNASeq are powerful tools to study methylation variation and transcription changes, no joint analysis with these two types of data have been reported in tissue engineering. Tissue engineering aims to develop biomimetic tissues that recapitulate biological, structural and functional characteristics of native tissue. Thus age-related changes have potential implications for the tissue engineering strategies used for enhancing musculoskeletal repair. In this study we evaluated and compared the methylome and transcriptome of chondrogenic, osteogenic and tenogenic engineered tissues derived from young and old human bone marrow derived MSCs in order to determine similar and distinct changes with ageing. In doing so we have identified areas for future research to improve functionality of ageing MSC derived engineered tissues.

## Materials and Methods

All chemicals are supplied by Sigma unless stated otherwise.

### Cell Culture and Differentiation

Human MSCs from young; n = 4 (21.8years±2.4SD) and old; n = 4 (65.5years±8.3SD) donors (Stem Cell Technologies, Grenoble, France and Promocell, Heidelberg, Germany), grown to passage 4 and each donor each differentiated into chondrogenic [25], osteogenic [26] and tenogenic [27] tissues as previously described and used in all subsequent experiments [28]. All tissue culture was undertaken in 5% oxygen and tissues harvested at 21 days (osteogenic) and 28 days (chondrogenic and tenogenic). All cells were purchased and thus ethical approval was not required.

### Validation of differentiation

Differentiation of chondrogenic and osteogenic engineered tissues was assessed by comparing to MSCs treated identically except with maintenance media (complete Dulbecco’s Modified Eagles Media (Gibco)) using histology, and quantitative real-time PCR (qRT-PCR). Calcium depositions were determined in osteogenic tissues using Alizarin red staining as previously described [29]. Chondrogenic pellets were paraffin embedded and 4 μm sections taken and further stained with Alcian Blue/Nuclear Fast Red. Tendon engineered tissues were fixed in 4% paraformaldehyde, longitudinally embedded in paraffin and 4μm sectioned on polylysine slides. Staining was undertaken with Masson’s Trichrome [30].

Transmission electron microscopy (TEM) of tendon tissues were performed by fixation in 2.5% glutaraldehyde in 0.1M sodium cacodylate buffer followed for 8 hours, followed by buffer washing procedure and second fixation and contrast stain with 0.1% osmium tetroxide for 90 minutes. Samples were stained with 8% uranyl acetate in 0.69% maleic acid for 90 minutes, dehydrated in ascending ethanol concentrations and embedded in epoxy resin. 60–90 nm sections were cut with a Reichert- Jung Ultracut on an ultramicrotome using a diamond knife, mounted on 200 mesh copper grids and stained with ‘Reynold’s Lead citrate’ stain for 4 minutes. Images were viewed in Philips EM208S Transmission Electron Microscope at 80k.

RNA was extracted from all assays and converted to cDNA to analyse lineage-specific gene expression markers using qRT-PCR relative to GAPDH [22]. All primer sequences are in S1 file.

### RNA isolation, library preparation for RNASeq and small RNASeq and sequencing

Total RNA was isolated using TRIzol (Invitrogen^™^ Life Technologies, Carlsbad, USA) [31] and purified using RNeasy spin columns with on-column DNase treatment (Qiagen, Crawley, UK). Sequencing used the Illumina HiSeq 2000 (Illumina, San Diego, USA) at 2 × 100-base pair (bp) paired-end sequencing with v3 chemistry for RNASeq. Multiplexed size selected small RNA library pools were sequenced on one lane of the Illumina HiSeq 2500 (Illumina, San Diego, USA) at 1x50 bp sequencing. Details are in S2 file and [24].

### RNA Data processing

The RNASeq data was processed as previously described [22, 24]. Concise details are in S2 file. Data was assessed using pairwise comparisons, correlation heatmaps and PCA plots and outliers removed accordingly. For RNASeq and smallSeq differentially expressed genes (DEGs) and transcripts were extracted by applying the threshold of false discovery rate (FDR) adjusted p-values < 0.05, generated using the Benjamini and Hochberg approach [32] and a 1.4 log_2_ fold change (Log_2_FC). Sequence data have been submitted to National Centre for Biotechnology Information Gene Expression Omnibus (NCBI GEO) under Array Express accession number E-MTAB-3427.

### Genomic DNA isolation, bisulphite treatment and methylation profiling

Genomic DNA was extracted using the SureSelect gDNA Extraction Kit (Agilent, Santa Clara, USA) according to manufacturer’s instructions, 500 ng of genomic DNA was then bisulphite converted using the EZ-96 DNA Methylation Kit (Zymo Research, Irvine, USA). DNA methylation profiling of the samples was carried out by Cambridge Genomic Services (Cambridge, UK), using the Illumina Infinium HumanMethylation450 Beadchip array (Illumina, Inc., San Diego, USA).

### Methylation data processing

GenomeStudio (Illumina Inc., San Diego,USA) was used to extract the raw data. GenomeStudio provides the methylation data as β values: β = M/(M + U) (M represents the fluorescent signal of the methylation probe; U represents the signal of the un-methylated probe). β values range from 0 (no methylation) to 1 (100% methylation). The raw methylation data was processed using R (version 3.0.1) and the Watermelon package (version 2.12) as has been previously described [24, 33]. Probes with a detection P value > 0.01 were removed. Age-related differential methylation was defined as Benjamini—Hochberg corrected P value [32] < 0.01 or <0.05 (differentially methylated loci (DML) and gene/CpG island/promoter respectively) and a mean methylation difference (Δ β score) ≥0.15 (15%), as previously reported [34].

### Functional analysis of transcriptomic and methylation data

To determine gene ontology, functional analyses, networks, canonical pathways and protein-protein interactions of age-related differentially expressed genes and methylated genes we performed analyses using Panther Classification System [35] and the functional analysis and clustering tool from the Database for Annotation, Visualisation, and Integrated Discovery (DAVID bioinformatics resources 6.7) [36] (using expressed genes as a reference), Ingenuity Pathway Analysis (IPA) [37]. Targetscan v6.2 was used to identify potential miR [38].

### Relative gene expression using real-time polymerase chain reaction (*qRT-PCR)*

qRT-PCR was undertaken on engineered tissues from similarly sourced MSCs at P4 from an independent cohort from those used for the RNA-Seq analysis young; n = 3 (22.2years±2.3SD) and old; n = 3 (64.8years±6.6SD). Primers were either validated in previous publications [23, 39] and supplied by Eurogentec (Seraing, Belgium) or designed and validated commercially (Primer Design, Southampton, UK). Steady-state transcript abundance of potential endogenous control genes was measured in the RNA-Seq data. Assays for four genes—Glutaraldehyde dehydrogenase (GAPDH), ribosomal protein 13 (RPS8), ribosomal protein 13 (RPS13), and ribosomal protein 16 (RPS16) were selected as potential reference genes as their expression was unaltered. Stability of this panel of genes was assessed by applying a gene stability algorithm [40]. RPS8 was selected as the most stable endogenous control gene. For miRs cDNA synthesis was performed using 100 ng RNA and miRscript RT kit II (Qiagen, Crawley, UK) according to the manufacturer’s protocol. qRT-PCR analysis was performed using miRScript SybrGreen Mastermix (Qiagen, Crawley, UK) using Rnu-6 as the endogenous control. Relative expression levels were calculated by using the 2−ΔCt method [40]. Data were analysed statistically using GraphPad Prism 6 (GraphPad Software, San Diego, CA, USA) following normality testing using a Mann-Whitney test at a 0.05 level of significance.

## Results

### Characterisation of engineered tissues

**Fig 1:**
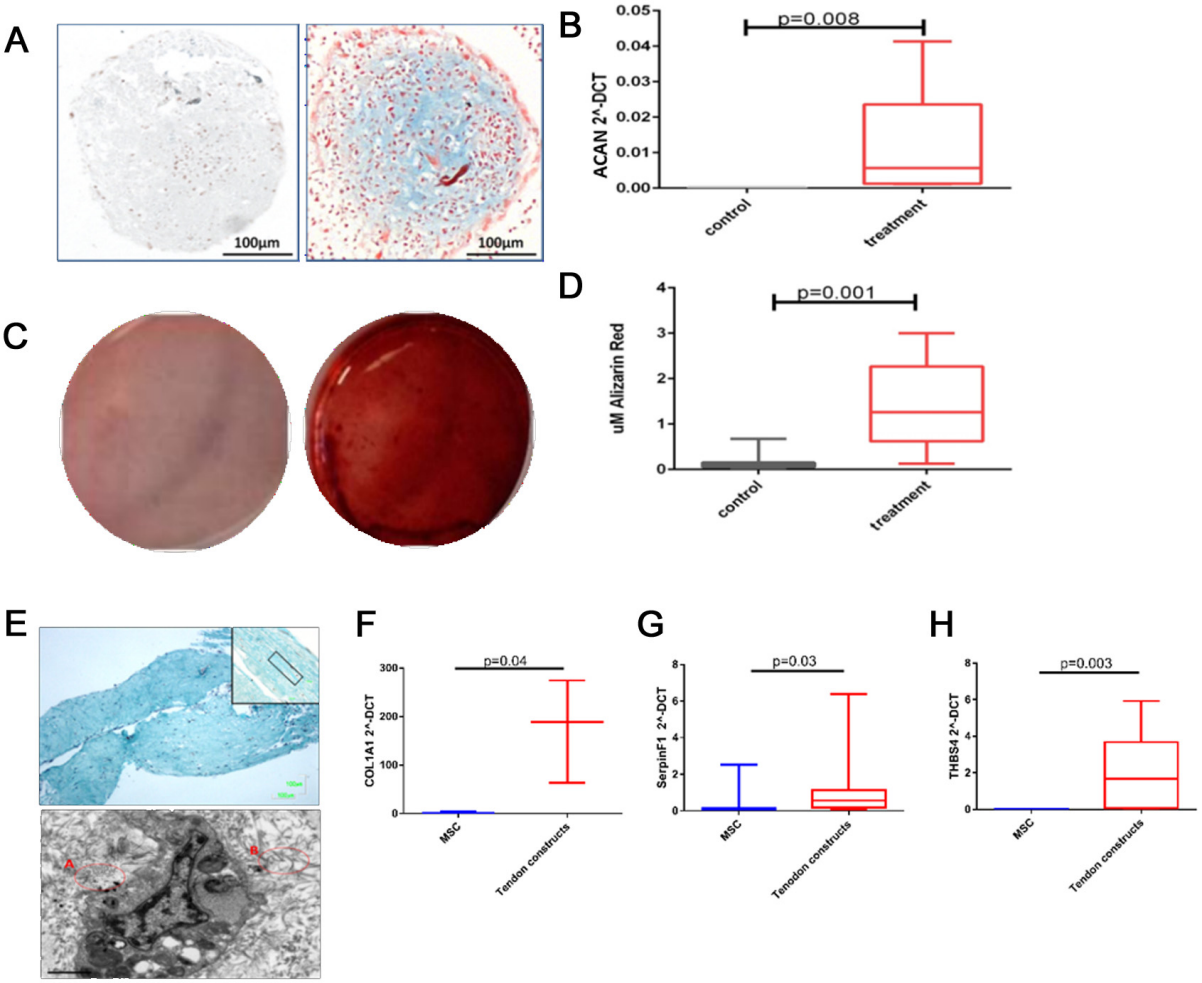
Histochemical and gene expression analysis of chondrogenic, osteogenic and tenogenic lineage differentiation for MSCs. Images are representative of all experiments. a; MSC pellets cultured in control or chondrogenic media were fixed and stained with Alcian Blue (scale bar 100μm, young donor shown) b;. Gene expression of aggrecan following chondrogenic differentiation, young and old donors combined. Data are represented as 2^-DCT compared with GAPDH. Box and whisker plots represent the median and 25–75 percentiles. Statistical evaluation was undertaken using Mann Whitney-U test (n = 6). c; Osteogenic differentiation from MSCs was confirmed with Alizarin Red S staining at day 21 to visualize mineralized bone matrix following extraction of the calcified mineral from the stained monolayer at low pH (young donor shown). d; Box and whisker plot showing quantitative results of Alizarin red staining of all donors, statistical significance Mann-Whitney-U test p<0.001 (n = 12). e; Histology images of a tendon engineered tissue (young donor shown) made from MSCs stained with Masson’s Trichrome to identify collagenous matrix. Image was captured at x4 magnification and x10 magnification inset (upper image); scale bar is 100μm. Example of more organised areas of collagen is marked on the inset image. Lower image depicts ultrastructural analysis using scanning transmission electron microscopy. The presence of aligned extracellular collagen fibrils (A) and less organised collagen (B) are inset in red; scale bar is 1μm. Tenogenic differentiation was also evaluated with using gene expression of f; COL1A1, g; SERPINF1 and h; THBS4. Data from all donors are represented as 2^-DCT compared with GAPDH. Statistical evaluation was undertaken using Mann Whitney-U test (n = 8).

To evaluate chondrogenesis markers of chondrocytes were assessed; Alcian Blue staining for glycosaminoglycans and aggrecan gene expression. There was an increase in Alcian Blue staining and aggrecan (ACAN) expression (Fig 1A and 1B). Osteogenesis was evaluated with Alizarin Red staining. There was a significant increase in staining with Alizarin Red both visually and using quantitative analysis (Fig 1C and 1D) demonstrating osteogenesis. Tenogenic differentiation was evaluated histologically using Masson’s Trichrome staining indicating areas of organised and disorganised collagen fibril formation within the tissues which were confirmed with TEM and gene expression of COL1A1 (Fig 1E and 1F), Serpin peptidase inhibitor F (SERPINF1) (Fig 1G) and thrombospondin 4 (THBS4) (Fig 1H) [41]. There were no age-related differences in the differentiation markers measured (data not shown).

### Overview of RNASeq and smallSeq data

For the RNASeq data an average of 68.53 million pairs of 100-bp paired-end reads per sample was generated that aligned to the human reference sequence. Based on data variation assessment one young and one old sample from chondrogenic were classed as outliers and removed from subsequent data analysis. Mapping results are summarised in the S3 file. Of the 63,152 human genes, between 39.9% and 47% had at least one read aligned [42]. This is similar to the output of other RNA-Seq studies [43]

In the smallSeq data an average of 12.2 million 50bp single-end reads was generated. This represented an average of 44% of reads mapped. Many of the 4206 human small RNAs were mapped with at least one read; 21.5–38.5% within all samples. Mapping results are summarised in S4 file and are similar to other small RNASeq studies [44, 45]. Reads were used to estimate small RNA transcript expression of all samples using FPKM in order to identify the most abundant miRs and small nucleolar RNAs (snoRNAs). S5 file demonstrates the expression levels of the entire data set and highlights the top 10 highly expressed small RNAs genes within each class.

### Identification of differentially expressed genes and differentially spliced genes using RNASeq

**Fig 2:**
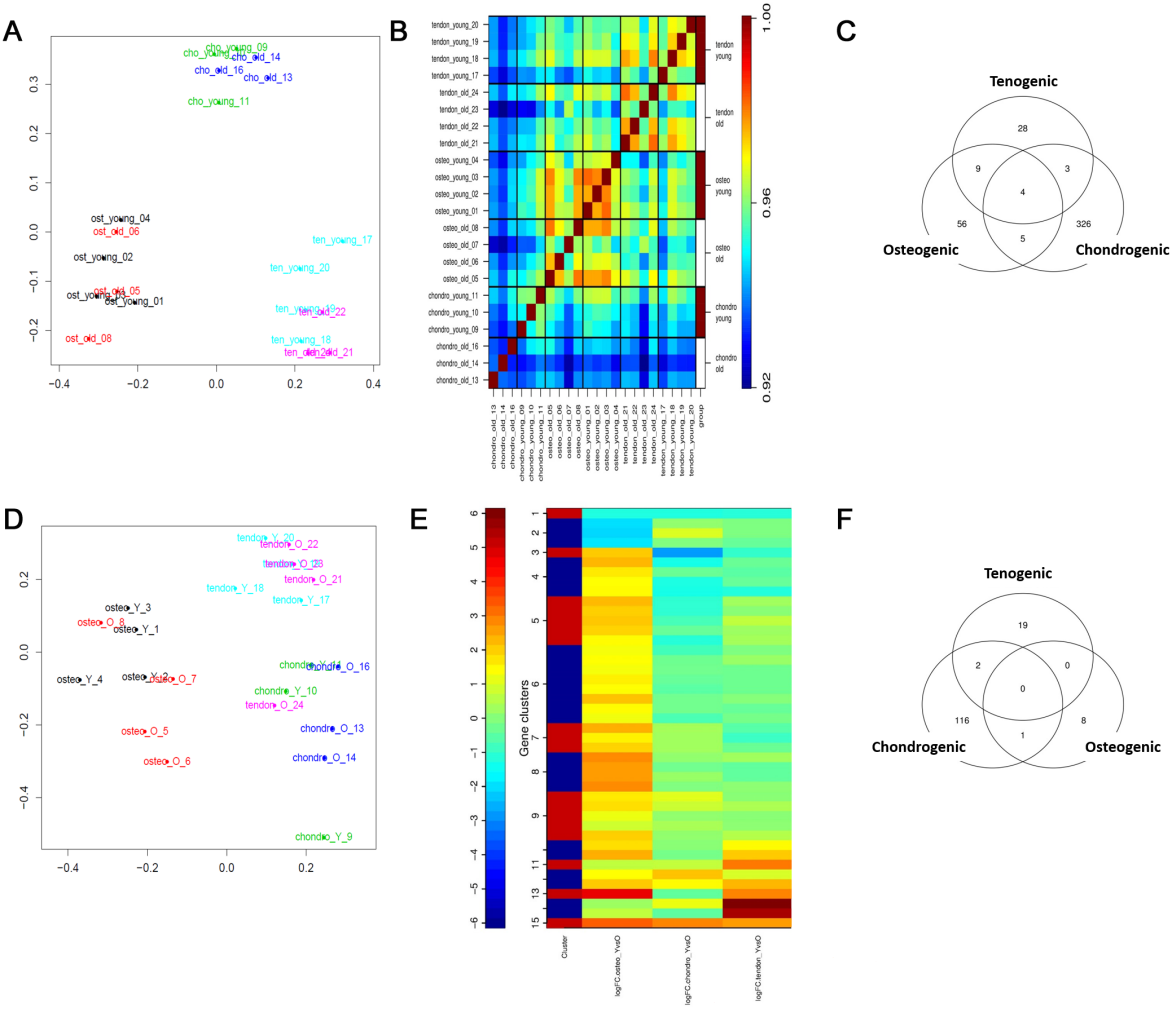
(A) A PCA plot of RNASeq data revealed the greatest variability in RNASeq data was tissue type. (B) Correlation heatmap of RNASeq data from chondrogenic (chondro), osteogenic (osteo) and tenogenic (tendon) engineered tissues derived from young and old MSCs. Samples from same tissue are more closely correlated than sample from different tissue. (C) Venn diagram showing the DE genes from RNASeq for chondrogenic, osteogenic tenogenic engineered tissues (D) PCA plot of small RNASeq data. (E) Correlation heatmap of age-related DE small RNAs in chondrogenic (logFCchondro_Y vs O), osteogenic (logFCosteo_YvsO) and tenogenic (logFCtendon_YvsO) engineered tissues using small RNASeq. (F) Venn diagram depicting DE transcripts from RNASeq from differential splicing analysis for chondrogenic, osteogenic tenogenic engineered tissues. Hierarchical clustering of the samples revealed significant age-related changes in expression in osteogenic and tenogenic but not chondrogenic engineered tissues. Analysis was undertaken using the filters ±1.4 log2 fold change, FDR<0.05.

For RNASeq a principal component analysis (PCA) plot (Fig 2A) of log_2_ gene expression data identified age-related biological variation within all engineered tissue groups. Hierarchical clustering using a sample to sample distance matrix identified clustering principally by engineered tissue type (Fig 2B).

Sets of age-related differentially expressed (DE) genes were identified including protein-coding RNA, long non-coding RNA (lnc), small nucleolar RNA (snoRNA), small nuclear RNA (snRNA), pseudogenes and miRs (Table 1) (±1.4 log2 fold change, FDR<0.05). There were 201, 67 and 32 chondrogenic, osteogenic and tenogenic age-related DE protein-coding genes respectively (Fig 2C). Table 2 represents the top 10 most differentially expressed up and down chondrogenic, osteogenic and tenogenic tissues. All DE genes are in S6 file. S7 file contains FPKM values for all samples, S8 file contains the MA plots for RNASeq and smallSeq.

**Table 1:** Differentially expressed RNAs in chondrogenic, osteogenic and tenogenic engineered tissues based on RNA class (±1.4 log2 fold change, FDR<0.05).

Engineered tissue Type	RNA class	Number differentially expressed	Number increased in old	Number reduced in old
**Chondrogenic**	protein coding	201	133	68
	Inc	28	20	8
	miR	5	2	3
	snoRNA	4	0	4
	snRNA	6	1	5
	pseudogenes	35	13	22
**Osteogenic**	protein coding	67	29	38
	Inc	2	2	0
**Tenogenic**	protein coding	32	21	11
	Inc	5	1	4
	miR	1	1	0

**Table 2:** Protein-coding genes with the highest and lowest fold changes for each engineered tissue type. A; chondrogenic, B; osteogenic, C: tenogenic.

**A. chondrogenic**			
**Gene Symbol**	**Entrez Gene Name**	**Fold Change**	**FDR**
NTF3	neurotrophin 3	8.61	0
SLPI	secretory leukocyte peptidase inhibitor	7.55	0.01
BEST2	bestrophin 2	7.4	0.01
CD14	CD14 molecule	6.97	0
ANKRD53	ankyrin repeat domain 53	5	0.01
IL18	interleukin 18	4.99	0
FBXO24	F-box protein 24	3.97	0.03
DAPK1	death-associated protein kinase 1	3.77	0
SLC7A2	solute carrier family 7, member 2	3.62	0
ALX1	ALX homeobox 1	3.5	0
SLC22A2	solute carrier family 22, member 2	-8.22	0.02
MAB21L2	mab-21-like 2	-8.13	0
KYNU	kynureninase	-7.98	0
UBE2QL1	ubiquitin-conjugating enzyme E2Q family-like 1	-7.97	0
CA2	carbonic anhydrase II	-8.01	0.01
EBF2	early B-cell factor 2	-7.7	0
DLGAP1	discs, large homolog-associated protein 1	-7.62	0.01
PKHD1L1	polycystic kidney and hepatic disease 1 -like 1	-7.37	0.03
PCDHA7	protocadherin alpha 7	-7.32	0.02
IZUMO1	izumo sperm-egg fusion 1	-7.06	0.04
**B. Osteogenic**			
ALX1	ALX homeobox 1	6.2	0
TGFA	transforming growth factor, alpha	6.18	0
HTR1F	5-hydroxytryptamine receptor 1F, G protein-coupled	5.14	0.05
DDIT4L	DNA-damage-inducible transcript 4-like	5.39	0
MKRN3	makorin ring finger protein 3	4.32	0.03
PITX2	paired-like homeodomain 2	4.22	0
PDZRN4	PDZ domain containing ring finger 4	4.19	0.02
SPARCL1	SPARC-like 1 (hevin)	3.83	0
IL1RL1	interleukin 1 receptor-like 1	3.84	0.02
PRLR	prolactin receptor	4.11	0.02
EPHA7	EPH receptor A7	-7.36	0
SLC6A15	solute carrier family 6, member 15	-7.34	0
IL13RA2	interleukin 13 receptor, alpha 2	-7.23	0.01
MAB21L2	mab-21-like 2	-6.46	0
THBD	thrombomodulin	-7	0
TNXB	tenascin XB	-6.89	0
NOVA1	neuro-oncological ventral antigen 1	-5.69	0.02
SULT1B1	sulfotransferase family, cytosolic, 1B, member 1	-5.57	0.04
CNTNAP4	contactin associated protein-like 4	-5.25	0.03
DSG2	desmoglein 2	-5.31	0
**C. Tenogenic**			
ALX1	ALX homeobox 1	7.67	0
MKRN3	makorin ring finger protein 3	4.41	0.03
HOXB7	homeobox B7	3.5	0
HOXB6	homeobox B6	3.24	0.02
PITX2	paired-like homeodomain 2	3.31	0.01
PLAT	plasminogen activator, tissue	2.68	0.02
TNIK	TRAF2 and NCK interacting kinase	2.32	0
HOXA3	homeobox A3	2.41	0.01
AHDC1	AT hook, DNA binding motif, containing 1	1.72	0.03
ZNF462	zinc finger protein 462	1.64	0
MAB21L2	mab-21-like 2	-6.85	0
NPTX1	neuronal pentraxin I	-6.79	0.03
THEGL	theg spermatid protein-like	-6.54	0
SRRM3	serine/arginine repetitive matrix 3	-5.07	0.01
MCF2L	MCF.2 cell line derived transforming sequence	-5.03	0.01
GPM6B	glycoprotein M6B	-4.89	0.03
SYT16	synaptotagmin XVI	-4.88	0.04
ELFN2	III domain containing 2	-4.76	0
HS3ST2	heparan sulfate 3-O-sulfotransferase 2	-4.75	0
EPHA7	EPH receptor A7	-4.18	0.03

Log2 fold-change and false discovery rate (FDR) (adjusted P value) were determined in edgeR. A logarithm to the base 2 of 8 is a linear fold-change of 3. Shown are the 10 genes with highest and lowest expression in tissues derived from young compared to old MSCs. Negative LFC is higher in old.

In total 94190±3005 chondrogenic, 116105 ±3008 osteogenic, and 113075±5346 tenogenic isoforms were detected (mean±standard deviation)). No isoforms were differentially expressed. However, using Cuffdiff to calculate changes in the relative splice abundances by quantifying the square root of the Jensen-Shannon divergence on primary transcripts with at least two isoforms, identified 119, 21 and 9 differential splicing events in chondrogenic, osteogenic and tenogenic tissues respectively (alternative splicing (AS)) (S9 file). These included small nucleolar RNAs, long non-coding RNAs and pseudogenes.

For the smallSeq PCA of log_2_ gene expression data indicated the age-effect was weak (Fig 2D and 2E). The greatest variability was due to engineered tissue type. There were no age-related DE small RNAs in chondrogenic tissues. In osteogenic tissues, there were 36 DE miRs (all reduced were derived from old MSCs) and three DE snoRNAs and in tenogenic engineered tissues three miRs were DE (Fig 2F, Table 3). The donor age-associated DE of several miRs in the osteogenic and tenogenic tissues was validated using qPCR (Table 4). Validated miRs were chosen based on our own and published data with regards to the relevance to the osteogenic- and tenogenic-related processes.

**Table 3:** Age-related differentially expressed small RNAs in osteogenic and tenogenic engineered tissues.

Tissue engineered type	Gene identification	Log fold change	FDR
Osteogenic	miR-887-5p	5.57	0.02
	miR-10a-3p	4.07	0.01
	miR-369-3p	3.48	0.00
	miR-651-5p	3.47	0.00
	miR-542-3p	3.27	0.00
	miR-450b-5p	3.25	0.00
	miR-188-5p	3.03	0.05
	miR-143-3p	3.03	0.02
	miR-1307-5p	2.92	0.01
	miR-145-3p	2.88	0.01
	miR-455-5p	2.78	0.00
	miR-487a-3p	2.70	0.00
	miR-376b-5p	2.69	0.01
	miR-148a-3p	2.67	0.04
	miR-450a-5p	2.61	0.00
	miR-4775	2.60	0.04
	miR-655-3p	2.58	0.01
	miR-495-3p	2.57	0.00
	miR-1185-2-3p	2.43	0.01
	miR-137	2.42	0.01
	miR-1185-1-3p	2.27	0.02
	miR-136-3p	2.24	0.02
	miR-340-5p	2.22	0.02
	miR-30a-5p	2.14	0.01
	miR-493-3p	2.07	0.00
	miR-889-3p	2.06	0.02
	miR-656-3p	2.06	0.02
	let-7i-3p	2.00	0.02
	miR-382-3p	2.00	0.01
	miR-140-5p	1.97	0.02
	miR-370-3p	1.97	0.01
	let-7i-5p	1.86	0.00
	miR-27b-3p	1.83	0.00
	miR-98-5p	1.82	0.03
	miR-21-3p	1.77	0.05
	miR-22-3p	1.40	0.02
	U44	-1.56	0.01
	SNORD65	-1.69	0.04
	SNORD126	-1.79	0.02
Tenogenic	miR-500a-5p	6.93	0.00
	miR-548j-5p	6.40	0.04
	miR-618	3.71	0.00

±1.4 log2 fold change, FDR<0.05

**Table 4:** The differential expression of several miRs was validated using qPCR.

		RNASeq		qPCR results					
Engineered tissue type	microRNA	Log_2_FC	q-value	Young	Old	2^-ΔCTlog_2_FC	p-value	SEM young	SEM old
Osteogenic	let-7	2.0	0.02	0.38	0	8.57	0.04	0.14	0
	miR-21	1.77	0.05	502.29	24.98	4.33	0.03	169.65	14.09
	miR-30	2.14	0.01	8.77	0.18	5.61	0	2.11	0.06
	miR-96	NS	NS	0.37	0	8.53	0.02	0.16	0
	miR-27	1.83	0.00	3.95	0.35	3.50	0.05	1.52	0.08
	miR-140	1.97	0.02	1.64	0.03	5.77	0	0.78	0.01
Tenogenic	miR-500	6.93	0.00	1.47	0.78	0.91	0.02	0.18	0.23
	miR-548	6.4	0.04	1.09	0.02	5.77	0.04	0.55	0

Relative expression levels were calculated by using the 2−ΔCt method. Log2 fold-change of 2-ΔCt values are shown for comparison. NS; not significant

Reproducibility of RNASeq results with an independent platform is high [22, 23]. Nevertheless we selected genes (mRNA and miRNA) DE and assessed their expression levels with qRT-PCR analyses for each engineered tissue type. There was good correlation between the deep sequencing analyses and qRT-PCR results (Table 5) reflecting the accuracy and reliability of deep sequencing analyses.

**Table 5:** Real-time polymerase chain reaction analysis of selected genes for each engineered tissue type revealed good correlation with RNA-Seq results.

Engineered tissue type	Gene	RNA-Seq Results			RT-PCR Results			
					Age		2^^-ΔCT^log_2_FC	p-value
		Differential expression	Significant Log_2_FC	q-value	Young	Old		
Chondrogenic	ALX1	lower old	3.53	0.01	0.11±0.05	0.63±0.48	-2.52	0.3
	COL2A1	higher old	-6.74	0.01	0.02±0.01	0.2±0.02	-3.30	0.01
	ACAN	NS	NA	NA	0.01±0.00	0.06±0.03	-2.58	0.1
	MAB21L2	higher old	-3.84	0	0.01±0.00	0.47±0.33	-5.55	0.02
	MMP16	higher old	-2.82	0.02	0.00±0.00	0.52±0.51	-9.02	0.05
Osteogenic	ALX1	lower old	6.26	0	0.01±0.04	0.4±0.06	-5.32	0.02
	HOXB6		3.38	0.03	0.14±0.01	0.02±0.01	2.81	0.06
	HOXB7		3.28	0	0.31±0.16	0.03±0.02	3.37	0.05
	PITX2		4.6	0	0.03±0.01	0.01±0.00	1.58	0.03
	TGFA		5.64	0	0.42±0.28	0.02±0.01	4.39	0.03
Tenogenic	ALX1	lower old	7.6	0	164.67±29.02	0.26±0.11	9.31	0.02
	HOXB6		3.66	0.02	53.04±9.00	0.18±0.10	8.20	0.04
	HOXB7		3.77	0	55.2±10.1	0.25±0.10	7.79	0.02
	PITX2		3.51	0.01	22.68±2.85	0.32±0.12	6.15	0.04

Values for quantitative real-time polymerase chain reaction (qRT-PCR) are the mean ± standard error of relative expression levels normalised to expression of RPS8 (to two decimal places). Statistical significance was tested by using Mann-Whitney U test. q RT-PCR results are expressed as 2-ΔCt. Log2 fold-change of 2-ΔCt values are shown for comparison. ALX1; ALX homeobox 1, ACAN, aggrecan; COL2A1, collagen type 2 alpha 1; HOXB6; homeobox B6, HOXB7; homeobox B7, MAB21L2; MAB21-like 2, PITX2; paired-like homeodomain transcription factor 2, MMP16, matrix metalloproteinase 16; TGFA; transforming growth factor alpha. NS; not significant.

### Gene ontology (GO) and IPA analysis of DEGs and AS genes

**Fig 3:**
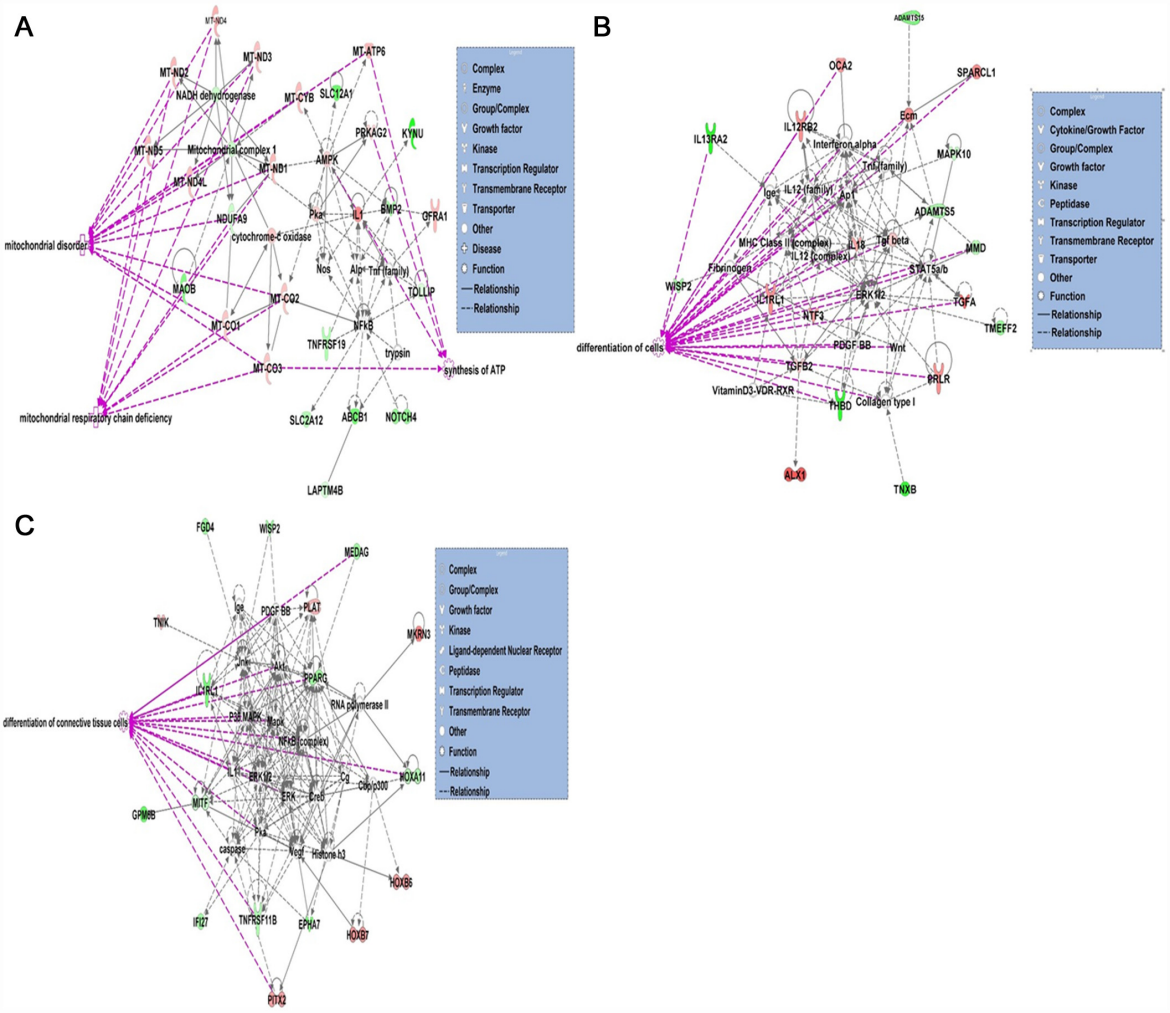
Top scoring networks. Networks derived from the DE genes with age-related different abundance identified the top network for each chondrogenic, osteogenic and tenogenic tissues with scores of 44, 41, and 42 respectively. These related to developmental disorders, hereditary disorders and metabolic disease for chondrogenic (A), cellular growth and proliferation, cell development and morphology for osteogenic (B) and embryonic and organismal development for tenogenic (C). Green nodes, increased expression in old; red nodes, lower expression in old; white nodes, genes not differentially expressed with age. Intensity of colour is related to higher fold-change. Legend to the main features in the networks is shown. Significant functions related to chondrogenic included mitochondrial disorders (p = 5.2e^-29^) and mitochondrial respiratory chain deficiency (p = 1.6e^-17^), for osteogenic include differentiation of cells (p = 4.5e^-5^) and for tenogenic include differentiation of connective tissue cells (p = 9.5e^-5^). These are highlighted in purple.

For each engineered tissue type age-related DEGs (adjusted P<0.05 and 1.4 log_2_ fold change) were analysed in DAVID. Significant annotations included shared terms ‘glycoprotein’ and ‘extracellular matrix’ (ECM) for chondrogenic and osteogenic. In addition the terms ‘growth factor’ and ‘secreted’ were identified for chondrogenic and osteogenic respectively. For tenogenic ‘developmental protein’ and ‘homeobox’ were significantly enriched. The DEGs were next input into IPA. This inferred the nature of the engineered tissue protein-coding transcript networks was predominantly for the functions ‘cell death and survival’, ‘cell morphology’, and ‘cell growth and proliferation’. The canonical pathways significantly identified from the gene sets are shown in Table 6. The top networks identified for each engineered tissue type were ‘developmental disorders, hereditary disorders and metabolic disease’ for chondrogenic (Fig 3A); ‘cellular growth and proliferation, cell development’ and ‘morphology’ for osteogenic (Fig 3B); and ‘embryonic and organismal development’ for tenogenic (Fig 3C).

**Table 6:** The top canonical pathways. Pathways from the IPA knowledge base that involve DE (adjusted P<0.05 and 1.4 log_2_ fold change) protein coding genes differentially expressed in tissues derived from young compared to old MSCs; chondrogenic, tenogenic and osteogenic.

Engineered tissue type	Ingenuity Canonical Pathways	-log(p-value)	Ratio
Chondrogenic	Mitochondrial Dysfunction	1.01E+01	7.94E-02
	Oxidative Phosphorylation	9.37E+00	1.00E-01
	GDNF Family Ligand-Receptor Interactions	3.48E+00	7.04E-02
	Hepatic Fibrosis / Hepatic Stellate Cell Activation	2.14E+00	2.99E-02
	Tryptophan Degradation	2.00E+00	1.11E-01
Osteogenic	IL-12 Signalling and Production in Macrophages	3.08E+00	2.88E-02
	VDR/RXR Activation	2.76E+00	3.80E-02
	Hepatic Cholestasis	2.70E+00	2.26E-02
	Hepatic Fibrosis / Hepatic Stellate Cell Activation	2.50E+00	1.99E-02
	Pancreatic Adenocarcinoma Signalling	2.37E+00	2.78E-02
Tenogenic	PPAR Signalling	3.38E+00	3.19E-02
	LPS/IL-1 Mediated Inhibition of RXR Function	2.33E+00	1.38E-02
	IL-6 Signalling	1.85E+00	1.72E-02
	Type II Diabetes Mellitus Signalling	1.85E+00	1.71E-02
	HMGB1 Signalling	1.83E+00	1.67E-02

The -log(p-values) were calculated by Fisher's exact test right-tailed.

**Fig 4:**
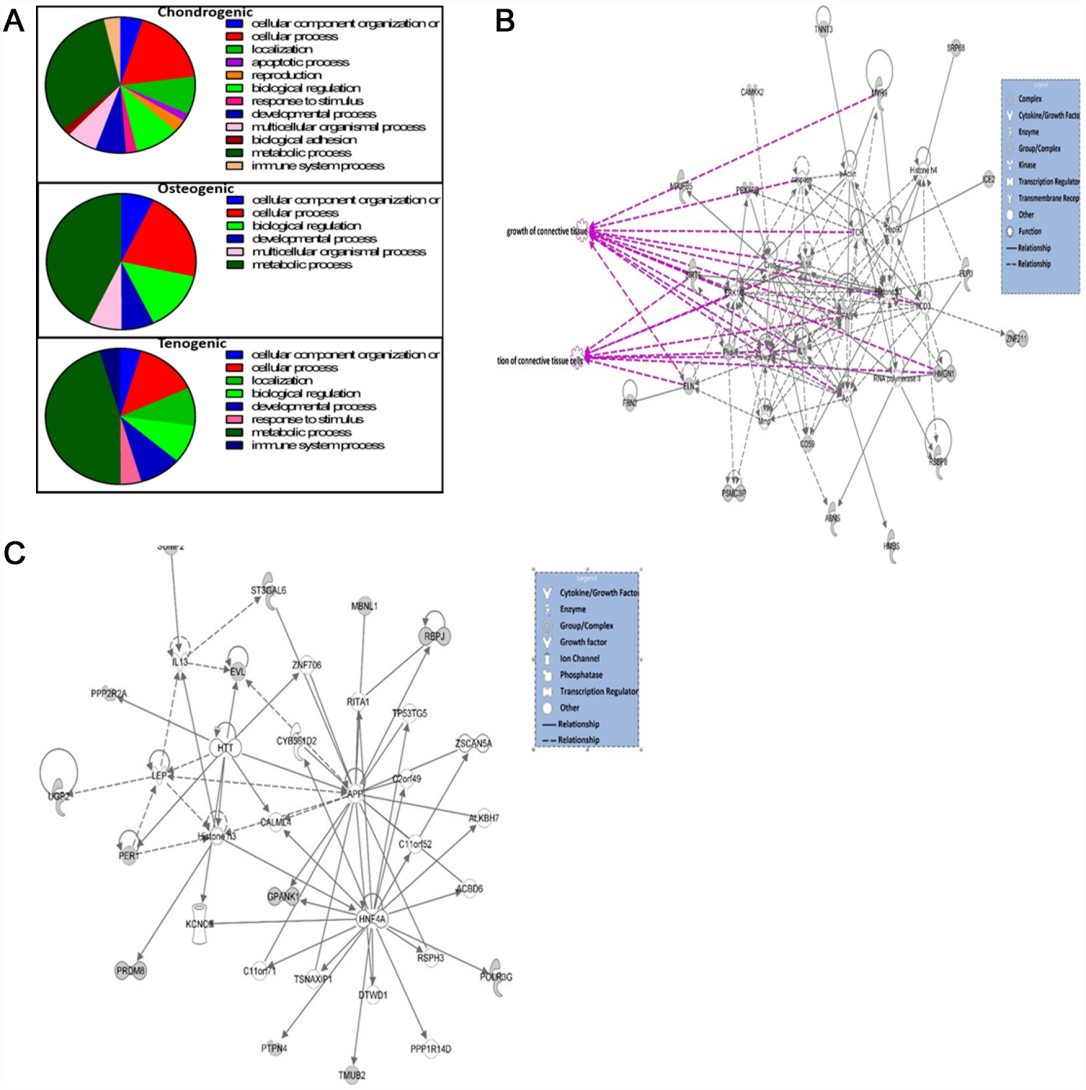
Bioinformatics analysis of AS in engineered tissues. A. Pie charts depicting biological process gene ontology of DE genes in ageing using PANTHER. Genes were demonstrated as DE with ±1.4 log2 fold change, FDR<0.05. B. The top scoring IPA derived network for significant AS genes in chondrogenic tissues. This related to’ cell death and survival, cellular compromise and connective tissue disorders’. Significant functions related to the network are overlaid; growth of connective tissue (p = 2.48E-08) and proliferation of connective tissue (p = 6.77E-07). C. The top scoring IPA derived network for significant AS genes in tenogenic tissues was ‘cell to cell signalling and interaction, cell morphology, function and maintenance’. Key to the main features in the networks is shown. Grey nodes were those identified as significant from the AS gene dataset, white nodes genes not in dataset.

GO analyses using PANTHER indicated enrichment in genes associated principally with metabolic processes in all engineered tissue type genes undergoing AS (Fig 4A). The chondrogenic and tenogenic AS gene sets was then analysed with IPA. For chondrogenic tissues the top pathways identified included cell death and survival (p = -2.96E-02- 5.42E-05), cellular compromise (p = 2.54E-02-5.42E-05), organismal survival (p = 2.96E-02-5.35E-04) and tissue development (p = 2.54E-02-6.04E-04). The top network identified was cell death, survival, cellular compromise, connective tissue disorders with a score of 40. Fig 4B shows this network with some significant functions overlaid; connective tissue (p = 2.48E-08) and proliferation of connective tissue (p = 6.77E-07). For tenogenic top pathways identified included carbohydrate metabolism (p = 1.73E-02-7.29E-04), lipid metabolism (p = 2.95E-02-7.29E-04), cellular function and maintenance (p = 3.65E02-7.29E-04) and connective tissue development and function (p = 3.3E-02-7.29E-04). The principal network involved cell to cell signalling and interaction, cell morphology, function and maintenance (score 36) (Fig 4C).

### Gene pairing analysis of DE miRs and DE RNAs

The expression patterns of DE miRs and mRNA were further analysed using IPA by investigating opposite fold-change direction (up/down or down/up), following the canonical miR-mRNA target expression paradigm with moderate to high confidence. Potentially relevant miR-mRNA signatures involved in the age-related changes were identified; 16 for osteogenic and one for tenogenic (S10 file). Using PANTHER the mRNA in which related miRs were identified in osteogenic tissues were enriched in the cellular components ECM (52% of genes) and enriched for the functions ‘binding (44%).

### Comparison of the DNA methylome in ageing MSCs

**Fig 5:**
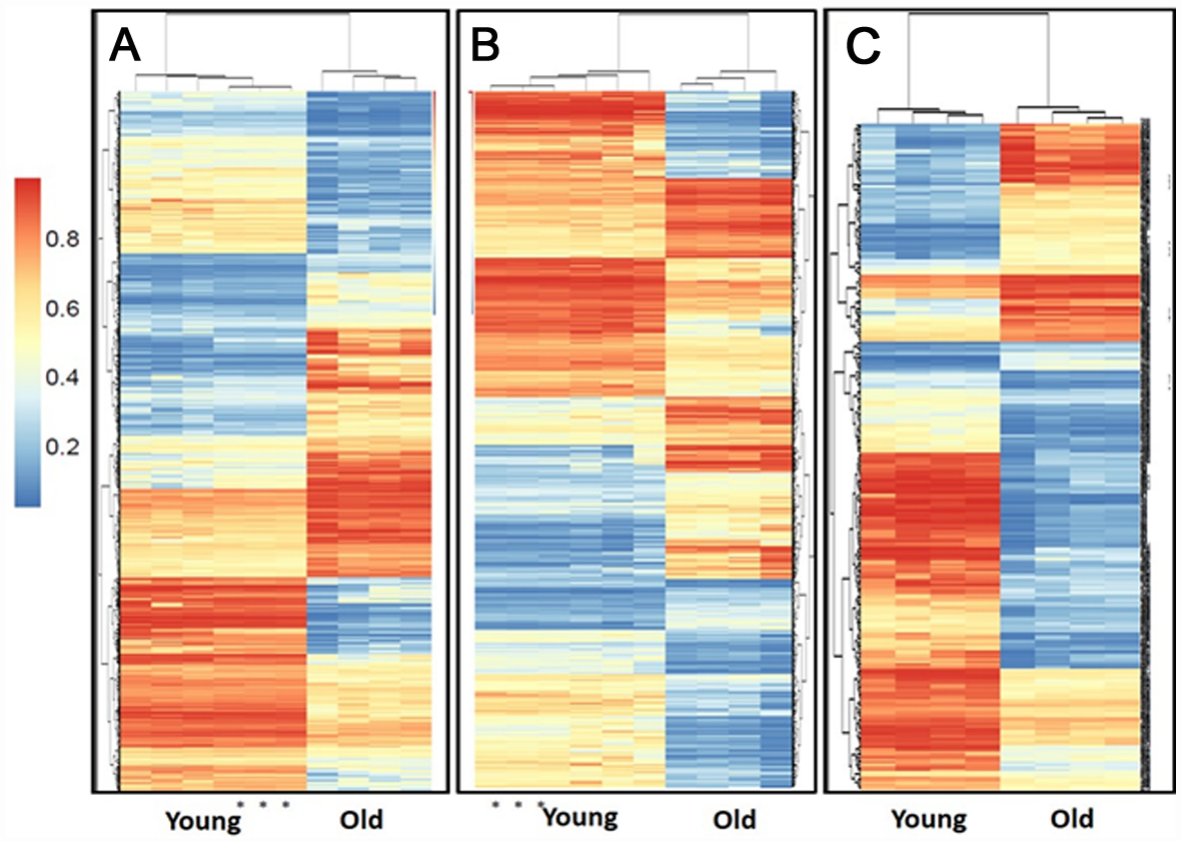
Heatmap showing the unsupervised clustering of the DMLs between the young (n = 4) and old (n = 4) chondrogenic (A), osteogenic (B) and tenogenic (B) engineered tissues. For chondrogenic young and osteogenic young a sample was run in triplicate technical replicates. DMLs were defined as at least a 10% difference in methylation between the two groups, and an FDR-corrected P value of <0.05. The dendogram at the top shows the clustering of the samples and the dendogram to the side show clustering of the loci. The methylation scale is shown at the left of the heatmap (1 = 100% methylation, 0 = no methylation).

Unsupervised hierarchical clustering revealed that young and old samples are distinguished by their DNA methylome in all engineered tissue types (Fig 5). Technical triplicate replicates were included for a single donor for chondrogenic and osteogenic young donors and correlation was excellent. Significant age-related differentially methylated loci (DML), both tissue specific and common CpGs, were identified in all engineered tissue types (Table 7). S11 file contains all DML for each engineered tissue type and S12 file identifies these at the site, promoter, gene and CpG island level. In all engineered tissue groups and regions hypomethylation in old samples was dominant.

**Table 7:** Number of age-related differentially methylated loci (DML), genes, CpG islands and promoters.

Engineered tissue type	Region	Total number DE	Number hypomethylated in old	Number hypermethylated in old
Chondrogenic	DML	609	402	207
	Gene	12	7	5
	CpG Island	58	43	15
	Promoter	17	12	5
Osteogenic	DML	507	367	140
	Gene	11	8	3
	CpG Island	38	32	6
	Promoter	15	12	3
Tenogenic	DML	157	122	45
	Gene	1	0	1
	CpG Island	314	300	14
	Promoter	30	27	3

Significance was defined as Benjamini—Hochberg corrected P value < 0.01 (DML) or <0.05 (gene, CpG island, promoter) and a mean methylation difference (Δ β score) ≥0.15.

Gene ontology analysis of genes containing DML indicated age-related enrichment for all engineered tissue types in skeletal system morphogenesis, regulation of cell proliferation and regulation of transcription.

To identify the canonical pathways, biological function, and networks that were affected by the differentially methylated genes, we used IPA analysis. Results suggest that dynamic epigenetic modifications may occur in genes associated with a number of shared and distinct pathways dependent upon engineered tissue type. The top 10 genes with increased and decreased methylation levels based on Beta values (methylation difference) are listed in Table 8.

**Table 8:** Top 10 annotated genes with increased and decreased methylation.

Engineered tissue type	Symbol	Gene Name	Beta	Location	Type(s)
chondrogenic	HOXA5	homeobox A5	-0.67	Nucleus	transcription regulator
	HAND2	heart and neural crest derivatives expressed 2	-0.66	Nucleus	transcription regulator
	mir-548	microRNA 548c	-0.63	Cytoplasm	microRNA
	SMTNL1	smoothelin-like 1	-0.60	Cytoplasm	other
	LAMA1	laminin, alpha 1	-0.60	Extracellular Space	other
	SHANK2	SH3 and multiple ankyrin repeat domains 2	-0.58	Plasma Membrane	other
	EMX2	empty spiracles homeobox 2	-0.57	Nucleus	transcription regulator
	GAPT	GRB2-binding adaptor protein, transmembrane	-0.57	Other	other
	USP28	ubiquitin specific peptidase 28	-0.57	Nucleus	peptidase
	SAMD12	sterile alpha motif domain containing 12	-0.53	Other	other
	SLC12A7	solute carrier family 12, member 7	0.64	Plasma Membrane	transporter
	LRBA	LPS-responsive vesicle trafficking	0.64	Cytoplasm	other
	HOXB4	homeobox B4	0.68	Nucleus	transcription regulator
	RUNX3	runt-related transcription factor 3	0.70	Nucleus	transcription regulator
	PITX2	paired-like homeodomain 2	0.71	Nucleus	transcription regulator
	HOXA11-AS	HOXA11 antisense RNA	0.72	Other	other
	mir-10	microRNA 100	0.73	Other	microRNA
	HOXB7	homeobox B7	0.74	Nucleus	transcription regulator
	EMX2OS	EMX2 opposite strand/antisense RNA	0.76	Other	other
	TBX15	T-box 15	0.91	Nucleus	transcription regulator
osteogenic	HOXA5	homeobox A5	-0.77	Nucleus	transcription regulator
	HOXA2	homeobox A2	-0.76	Nucleus	transcription regulator
	LAMA1	laminin, alpha 1	-0.69	Extracellular Space	other
	PARP4	poly (ADP-ribose) polymerase family, member 4	-0.67	Cytoplasm	enzyme
	SIX2	SIX homeobox 2	-0.65	Nucleus	transcription regulator
	PRRX1	paired related homeobox 1	-0.62	Nucleus	transcription regulator
	CPNE4	copine IV	-0.62	Cytoplasm	other
	GAPT	GRB2-binding adaptor protein, transmembrane	-0.62	Other	other
	MIR548F5	microRNA 548c	-0.61	Cytoplasm	microRNA
	USP28	ubiquitin specific peptidase 28	-0.58	Nucleus	peptidase
	BMX	BMX non-receptor tyrosine kinase	0.57	Cytoplasm	kinase
	DMRT2	doublesex and mab-3 related transcription factor 2	0.57	Nucleus	other
	TBX18	T-box 18	0.58	Nucleus	transcription regulator
	EPB41L5	erythrocyte membrane protein band 4.1 like 5	0.60	Plasma Membrane	other
	SLC12A7	solute carrier family 12, member 7	0.61	Plasma Membrane	transporter
	HOXB7	homeobox B7	0.67	Nucleus	transcription regulator
	HOXA11AS	HOXA11 antisense RNA	0.68	Other	other
	MIR10A	microRNA 100	0.71	Other	microRNA
	EMX2OS	EMX2 opposite strand/antisense RNA	0.78	Other	other
	PITX2	paired-like homeodomain 2	0.86	Nucleus	transcription regulator
	TBX15	T-box 15	0.89	Nucleus	transcription regulator
tenogenic	HOXA5	homeobox A5	-0.74	Nucleus	transcription regulator
	LAMA1	laminin, alpha 1	-0.69	Extracellular Space	other
	HOXA3	homeobox A3	-0.69	Nucleus	transcription regulator
	PARP4	poly (ADP-ribose) polymerase family, member 4	-0.65	Cytoplasm	enzyme
	PRRX1	paired related homeobox 1	-0.59	Nucleus	transcription regulator
	CPNE4	copine IV	-0.57	Cytoplasm	other
	HOXB2	homeobox B2	-0.50	Nucleus	transcription regulator
	EMX2	empty spiracles homeobox 2	-0.49	Nucleus	transcription regulator
	PHACTR1	phosphatase and actin regulator 1	-0.46	Cytoplasm	other
	GRIK3	glutamate receptor, ionotropic, kainate 3	-0.45	Plasma Membrane	ion channel
	KHDRBS3	KH domain containing, signal transduction associated 3	0.55	Nucleus	other
	BMX	BMX non-receptor tyrosine kinase	0.55	Cytoplasm	kinase
	HOXB4	homeobox B4	0.56	Nucleus	transcription regulator
	RUNX3	runt-related transcription factor 3	0.60	Nucleus	transcription regulator
	TBX5	T-box 5	0.63	Nucleus	transcription regulator
	LRBA	LPS-responsive vesicle trafficking	0.72	Cytoplasm	other
	mir-10	microRNA 100	0.72	Other	microRNA
	EMX2OS	EMX2 opposite strand/antisense RNA	0.78	Other	other
	PITX2	paired-like homeodomain 2	0.80	Nucleus	transcription regulator
	TBX15	T-box 15	0.89	Nucleus	transcription regulator

**Fig 6:**
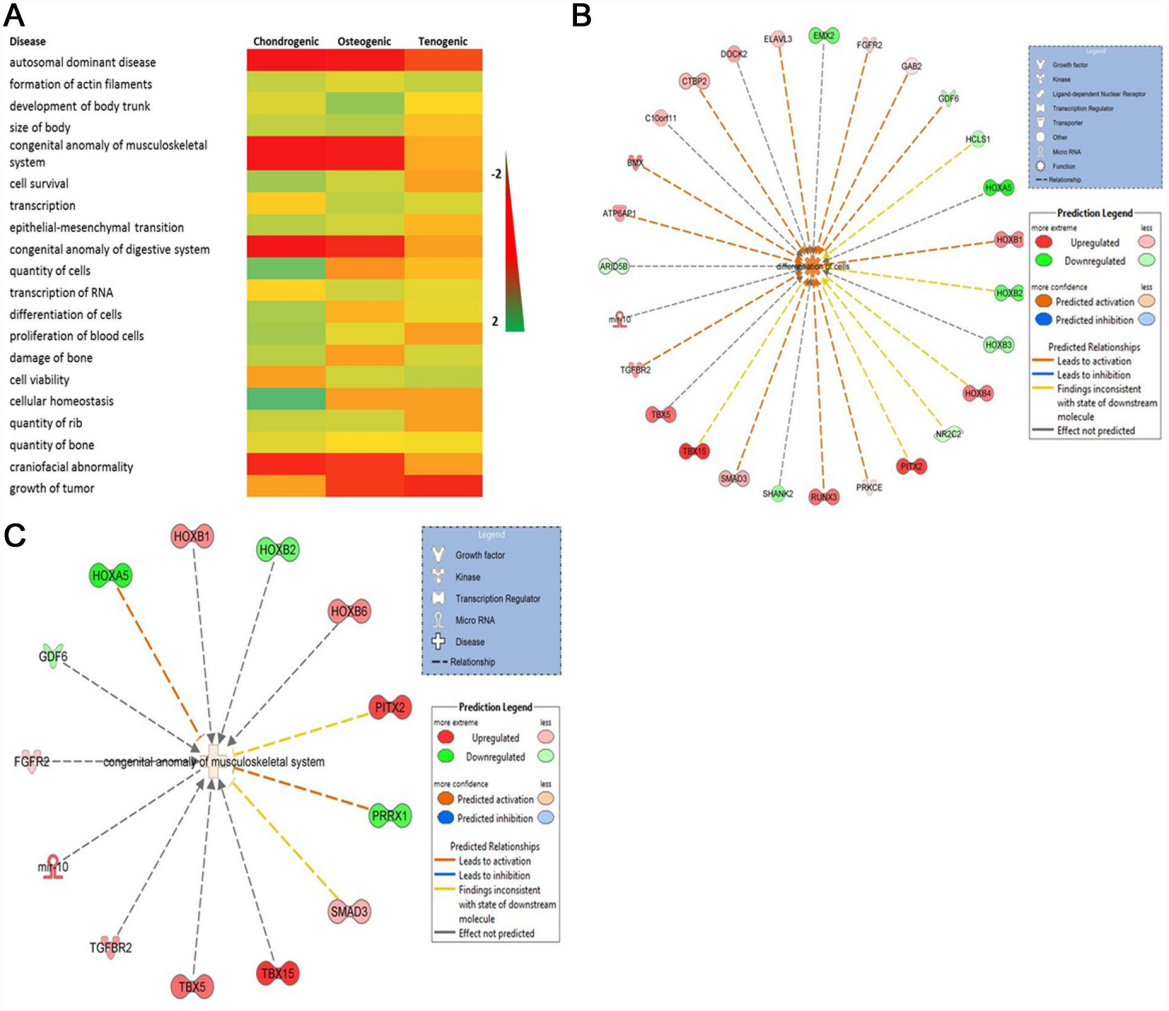
Diseases and biological functions identified from the sets of DM loci input into IPA for chondrogenic, osteogenic and tenogenic engineered tissues. A. Heatmap of the top 20 diseases and biological functions identified using IPA comparison analysis with significant activation z scores (infers the activation state of regulation). Scale relates to activation Z scores were green is a positive activation z-score (activated) and red is a negative score (inhibited). B. A cell differentiation network was identified in all engineered tissue types. The network shown includes DM genes identified in tenogenic tissues. C. The network ‘congenital anomaly of the musculoskeletal system’ was activated in tenogenic tissues. In networks red genes relates to those hypomethylated and green hypermethylated in tissues derived from older MSCs.

Canonical pathways were analysed based on the ratio of input genes to the total number of reference genes in the corresponding pathways in the IPA knowledge bases. Fisher’s exact test was employed to calculate the P values to determine significant associations between the DM genes and the canonical pathways. The top five canonical pathways for each engineered tissue type are in Table 9. Then we used IPA comparison analysis to visualise downstream effects analysis results across each engineered tissue type simultaneously. This identified diseases and biological functions predicted to increase or decrease related to age-affected DML through functional scores (Fig 6A). Interestingly in all engineered tissue types the function ‘differentiation of cells’ was activated (Fig 6B) whereas the ‘cell survival’ network was only affected in chondrogenic and osteogenic tissues. The network ‘congenital anomalies of the musculoskeletal system’ was activated in tenogenic but inhibited in chondrogenic and osteogenic. The most significant network for each engineered tissue was ‘skeletal and muscular development and function’.

**Table 9:** The five significant canonical pathways related to changes in the methylation patterns for each tissues type. The log (p-value) of each pathway was determined using Fisher’s exact test. The ratios were calculated as the number of input molecules mapped to a specific pathway divided by the total number of molecules in the given pathway.

Engineered tissue Type	Ingenuity Canonical Pathways	-log(p-value)	Ratio
Chondrogenic	Hepatic Fibrosis	5.62E+00	8.96E-02
	mTOR Signalling	4.61E+00	8.25E-02
	Tight Junction Signalling	4.19E+00	8.38E-02
	Chronic Myeloid Leukemia Signalling	4.03E+00	1.08E-01
	IL-9 Signalling	3.80E+00	1.76E-01
Osteogenic	AMPK Signaling	3.49E+00	6.63E-02
	Neuropathic Pain Signalling In Dorsal Horn Neurons	3.40E+00	8.65E-02
	VEGF Family Ligand-Receptor Interactions	2.73E+00	8.54E-02
	Glutamate Receptor Signalling	2.61E+00	9.38E-02
	Human Embryonic Stem Cell Pluripotency	2.51E+00	6.47E-02
Tenogenic	TGFB Signalling	3.30E+00	4.60E-02
	Chronic Myeloid Leukemia Signalling	3.20E+00	4.30E-02
	Antiproliferative Role of TOB in T Cell Signalling	2.27E+00	7.69E-02
	Factors Promoting Cardiogenesis	2.16E+00	3.26E-02
	Hepatic Fibrosis	1.99E+00	1.99E-02

Next we wanted to determine to what degree the age-related gene expression differences among engineered tissue types are affected by epigenetic changes. The methylation of gene promoters and/or enhancers is known to correlate with decreased gene expression, contrastingly methylation within non-enhancer regions of the gene body correlates with increased gene expression [46]. Therefore for chondrogenic, osteogenic and tenogenic tissues we compared DEGs from RNASeq with location of DMLs and found 10, 13 and 4 genes identified in both data sets for comparison (Table 10).

**Table 10:** Summary of genes and DML correlating relationships for chondrogenic, osteogenic and tenogenic engineered tissues.

Engineered tissue type	Gene ID	DML (B value)	Methylation status in old	Log2fold change (RNASeq)	Gene expression status in old	Location of methylation	Promoter/enhancer/body	Data correlation
Chondrogenic	FAM134B	0.3	hypomethylated	-2.6	higher old	Body;TSS200;5UTR	body, enhancer	yes
	H19	0.2	hypomethylated	-6.8	higher old	Body	body	no
	HOXB7	0.7	hypomethylated	3.3	lower old	Body	body	yes
	IRS2	0.4	hypomethylated	2.0	lower old	Body	body	yes
	KYNU	0.1	hypomethylated	-8.0	higher old	Body	body	no
	LRCH2	-0.2	hypermethylated	-3.2	higher old	Body	body	yes
	MAB21L2	0.3	hypomethylated	-8.1	higher old	Body;1stExon;5UTR	body, promoter	yes
	MAPK10	0.4	hypomethylated	-2.9	higher old	5UTR;1stExon	promoter	yes
	MYO7A	0.3	hypomethylated	-3.0	higher old	TSS200;TSS200;TSS200	enhancer	yes
	TMEM186	0.3	hypomethylated	1.9	lower old	3UTR	body	yes
Osteogenic	MAB21L2	0.4	hypomethylated	-7.0	higher old	Body;3UTR	body	no
	TNXB	0.4	hypomethylated	-6.9	higher old	Body	body	no
	WISP2	0.1	hypomethylated	-3.1	higher old	TSS200	enhancer	yes
	NTNG1	-0.1	hypermethylated	-2.6	higher old	Body	body	yes
	TBX18	0.6	hypomethylated	-1.8	higher old	Body	body	no
	MACROD2	0.2	hypomethylated	2.6	lower old	Body;TSS1500	body, promoter	yes
	ITIH5	0.1	hypomethylated	3.0	lower old	Body	body	yes
	KIAA1244	-0.4	hypermethylated	3.2	lower old	enhancer	enhancer	yes
	HOXB7	0.7	hypomethylated	3.3	lower old	Body	body	yes
	HOXB6	0.5	hypomethylated	3.4	lower old	TSS1500	promoter	no
	OCA2	-0.6	hypermethylated	3.8	lower old	enhancer	enhancer	yes
	PITX2	0.9	hypomethylated	4.6	lower old	Body; TS1500	body, enhancer	yes
	MKRN3	-0.3	hypermethylated	4.7	lower old	TSS200; 5UTR	promoter	yes
Tenogenic	HOXA3	-0.7	hypermethylated	2.1	higher old	5UTR;TSS1500	promoter	no
	HOXB6	0.5	hypomethylated	3.7	higher old	TSS1500	promoter	yes
	MAB21L2	0.3	hypomethylated	-6.8	lower old	Body;5UTR, TSS1500	body, enhancer	no
	PITX2	0.8	hypomethylated	3.5	higher old	Body;5UTR, TSS1500	body, enhancer	yes

The 3'UTR is encompassed in the gene body. The promoter is classified as the 5'UTR up to 1500bp upstream of the start codon. TSS; transcription start site, enhancer; where probes are within identified enhancer regions.

## Discussion

Adult MSCs are an appealing source for cell-based treatment for musculoskeletal diseases and injury [47]. Our previous work in bone-marrow-derived MSCs using a systems biology approach demonstrated an altered phenotype in MSC ageing at a number of levels, implicating roles for inflammageing and mitochondrial ageing [48]. The changes identified represented novel insights into the ageing process, with implications for stem cell therapies in older patients.

Tissue engineering aims to develop biomimetic tissues that recapitulate biological, structural and functional characteristics of native tissue. However there is sparse global information available on the effect of donor age on engineered musculoskeletal tissues at the transcriptome and methylome level. Age-related changes have potential implications for the tissue-engineering strategies used for enhancing musculoskeletal repair. Furthermore the study of musculoskeletal ageing in bone, cartilage and tendon are usually undertaken in seclusion and it is frequently difficult to attain aged matched tissue samples in humans. Therefore we propose our approach as a potential model of musculoskeletal ageing that could be probed further to identify factors that may aid in recapitulation of a younger tissue phenotype.

It is known that the site of MSC extraction can affect cell behaviour [49] we therefore used MSCs derived from alveolar bone. Additionally, as low oxygen tension improves MSC vitality and metabolic state in culture [50] all MSCs and then subsequently tissues were cultured in 5% oxygen tension. Standard methods of engineered tissue characterisation were undertaken following chondrogenic and osteogenic differentiation.

Transcriptome profiling is a key method for functional characterization of cells and tissues. However one challenge of this study was the integration of the different types of data. We used gene ontology, network analysis and annotated of the pathways identified. However, due to limited information on the donors we were unable to integrate key environmental factors such as nutrition and other lifestyle features which can alter molecular measurements. Other potential limitations of the study include small sample size and significance threshold filtering which can affect the subsequent pathway/network analysis.

Epigenetic processes have been implicated in age-related musculoskeletal diseases such as osteoarthritis [21] and osteoporosis (reviewed [51]). This study identified a number of epigenetic molecular classes including small non-coding RNAs; small nucleolar RNAs, small cajal body RNA (scaRNAs), miRs and lncRNAs.

Our study found weak age-related effects on expression at the miR level with no DE small RNAs in chondrogenic engineered tissues. The low mapping in chondrogenic samples implies that RNA populations or fragments other than the targeted small RNA were the input material into the library prep workflows. Further investigation did indeed demonstrate that the low percentage of alignments to the small RNA reference dataset corresponds to a high mapping percentage to rRNA in chondrogenic samples. However the percentage of mapping to rRNA for old and young samples was similar resulting in a lower sequencing depth which may reduce the statistical power in differential expression analysis. This effect was roughly similar for the two sample groups. Therefore, the count values are usable values, though the statistical power may be weaker due to lower sequencing depth. Compared to tenogenic and osteogenic, no DE miRNA detected from chondrogenic tissue is best explained by either no existence of DE miRNA, or existing DE miRNA was not detected due to lack enough statistical power.

Among the miR expression of which was differentially expressed in the osteogenic tissues from adult and old donors, miRs with known function in bone biology were validated: let-7 [52], miR-21 [53], miR-30 [54], miR-96 [55], miR-27 [56], and miR-140 [57]. Interestingly, among predicted targets of these miRs are genes and proteins regulated in MSC from adult and old donors as shown. For example, MMP16, predicted target of miRs miR-27, miR-30 and miR-140, is an important protein regulating bone homeostasis through regulating osteocyte differentiation [58]. Other genes of interest, predicted to be target of more than one of the validated miRs, include members of the ADAMTS family, key to cartilage and bone homeostasis [59], interleukin 18 with important role in bone metabolism [60]. Several genes with a not yet established function in MSC or bone biology, however reported to be expressed in these cells or tissues have also been characterised as DE in this study and are predicted target genes of miRs here validated, for example desmoglein 2 [61].

Interestingly, the expression of all miRs in the osteogenic tissues was downregulated in tissues from older donors. This may be due to defective miRNA biogenesis machinery [62] or decreased ability of the MSCs from older donors to undergo the osteogenic differentiation pathway [63]. Interestingly, the lower levels of expression of the enzyme associated with miR production, Dicer, in MSCs have been associated with their decreased differentiation potential [62].

We have also validated DE of miRs: miR-500 and miR-548j in the tenogenic tissues from young and older donors. It has been shown that miRs may play a role in tendon homeostasis [64, 65], however little is still understood about the role of miRs in tenogenic differentiation or tendon homeostasis. Based on target prediction databases), miRs: mir-500 and miR-548j may be regulating processes associated with matrix remodelling which are important in both tendon formation and maintenance, as well as healing. Interestingly, miR-548j is predicted to target peroxisome proliferator-activated receptor gamma (PPARG), a gene differentially expressed in tenogenic tissues from young and older MSCs donors. PPARG has been shown to be involved in tendon healing [66] further indicating the potential involvement of miR-548j in tendon repair.

To summarise, we have validated DE of miRs and their predicted target genes in the osteogenic and tenogenic tissues from young and older donors that may be associated with the decreased function of MSC from older donors and of relevance to MSC-based therapies.

LncRNAs play important roles in age-related diseases. Evidence is emerging that lncRNAs affect the molecular processes that underlie age-associated phenotypes. LncRNAs modulate gene expression patterns at the transcriptional, post-transcriptional and post-translational level. They affect many cellular processes relevant to ageing biology such as proliferation, differentiation and senescence (reviewed [67]). We identified a catalogue of lncRNAs for further work to define their roles in musculoskeletal ageing as although studies suggest the majority are functional only a few established biological relevance [68]. In tenogenic tissues we identified XIST as having a reduced expression in older tissues. XIST, responsible for imprinting controls epigenetic changes through DNA methylation and declines in senescence; though its function in this is unknown [69].

In transcriptomic studies we used gene ontology and network analysis tools to study pathways affected by MSC donor age. However, there are a few interesting findings for the some individual genes. Chondrogenic tissues were the most affected engineered tissue type with age demonstrated by the number of DEGs, whilst tenogenic were the least age-affected engineered tissue. Whilst principally large ‘omics’ datasets are analysed using network analysis to understand the overall effects of expression changes in the tissue, there were a number of interesting findings at the individual gene level that warrant discussion. In chondrogenic tissues the most DE gene was neurotrophin-3 (NTF3); highly expressed in young. This was not reflected in DML across the gene. NTF3 is an important gene in arthritic processes within the joint [70], produced by inflammatory cells of the nervous system as well as connective tissue [71], with survival-promoting and trophic effects on chondrocytes [72]. There is also a down-regulation of NTF expression in chondrocytes in arthritis [73]. Age-related changes in mouse brain have also been reported [74]. In osteogenic and tenogenic tissues ALX homeobox-1 had the most reduced expression in old similar to ageing MSCs [48]. It is important in skeletal development and we previously demonstrated an increased expression in old tendon [23]. Along with Homeobox (Hox) B7 (lower in old) and Mab-21-like 2 (higher in old) it was DE in all engineered tissue types and ageing MSCs [48]. HOX genes have been implicated in ageing of tissues [75] including tendon [23]. Furthermore HOX genes are required for tissue appropriate regeneration [76] and may be involved in the timing of ageing [49]. HOX-mediated transcriptional memory may reduce stem cell-mediated tissue regeneration [77]. Therefore this has special relevance to tissue engineering and musculoskeletal repair in ageing marking them as an interesting gene for further work in tissue engineering using MSCs.

Pathway analysis identified similar age-related changes at the molecular and cellular function level from input DE genes for the functions ‘cell death and survival’, ‘cell morphology’, and ‘cell growth and proliferation’. This suggests that although the DEG may be different between engineered tissue types the age-related pathways involved at this level are similar. Interestingly in ageing MSCs we demonstrated age-related changes in gene profiles included differences in cell proliferation, signalling, function and maintenance suggesting an age-related loss in MSCs ability to respond to biological cues [48]. Thus these changes seem to have impacted on all classes of engineered tissues.

The top canonical pathways in chondrogenic and tenogenic tissues were related to oxidative stress similar to that previously identified in tenocytes exposed to extracellular glucose [78] and ageing chondrocytes [79]. Mitochondrial dysfunction and oxidative phosphorylation were the most significant in chondrogenic tissues similar to ageing MSCs [48]. One hallmark of ageing is mitochondrial dysfunction and alterations in redox balance could account for the observed reduction in cellular function associated with age [80]. The mitochondria represent an important source of cellular ROS and recent evidence suggests that age-related oxidative stress can disrupt normal physiological cell signalling pathways. Human chondrocytes isolated from older adult chondrocytes exhibited increased peroxiredoxin (PRX) hyperoxidation, particularly for mitochondrial PRXs, when compared to younger chondrocytes in a recent study. PRXs represent a key antioxidant system, and oxidative stress mediated hyperoxidation leads to inhibition of PRX function, and a reduction in antioxidant capacity. PRX hyperoxidation was associated with inhibition of downstream pro-survival signalling and up-regulation of pro-death signalling which led to chondrocyte cell death [79]. Additionally, oxidative stress mediated inhibition of pro-survival cell signalling has also been demonstrated in another recent study supporting the theory that high levels of oxidative stress, such that are observed in ageing tissues, could lead to oxidative stress mediated alterations in cell signalling and contribute to the ageing phenotype and the development of age-related disease such as osteoarthritis [81]. The current study also identified significant alterations in cell signalling pathways in chondrogenic tissues. Thus the use of older MSC donors for cartilage tissue engineering could result in increased oxidative stress within the engineered tissue which could alter normal physiological signal transduction which could have significant consequences for the synthesis and degradation of cartilage matrix components [82]. Peroxisome proliferator-activated receptor signalling (PPAR) are key regulators in various age-related processes related to oxidative stress and energy metabolism. PPAR signalling was the dominant pathway in tenogenic tissues. As PPAR signalling has roles in cell proliferation, differentiation and tissue remodelling [83], and these pathways were also identified through a network of DE genes, this could have detrimental effects on engineered tendon from older MSCs. Furthermore PPAR signalling affects the impairment in mitochondrial biogenesis demonstrated in OA chondrocytes [84].

For osteogenic tissues age-related changes in genes involved in VDR/RXR (vitamin D receptor (VDR)-9-cis-retinoic acid receptor (RXR)) were identified. The classical actions of vitamin D3 are through this signalling pathway facilitating transcription of genes important in bone for the expression of several proteins including osteopontin [85] and in osteoblasts transcription of nuclear factor-kappaB ligand (RANK-L); important for the activation and differentiation of the osteoclasts [86]. A change in VDR expression with ageing has been reported in rat bone [87] and a reduction in ageing mice osteoblasts [88]. A significant effect on cell differentiation and survival is evident following a reduction in VDR activity in bone. Furthermore a decrease in VDR may be partially responsible for increased levels of apoptosis in ageing osteoblasts [88], together with reduction in bone mineralization proteins; osteopontin and osteocalcin [89]. In osteogenesis from ageing MSCs there are alterations in osteocalcin expression with negative effects on proliferation and differentiation capacity of BMSCs in culture [6]. Another study demonstrated osteogenic potential of ageing MSCs was reduced as measured by Alizarin Red staining (which stains calcium deposits) [90]. Together these findings suggest that the reduced osteogenic potential of ageing MSCs could in part be due to a dysregulation of VDR/RXR signalling. The most significant canonical pathways related to changes in the methylation patterns for osteogenic tissues was active AMP-activated protein kinase (AMPK) signalling. AMPK is highly affected by age and may be a crucial cell signalling pathway that regulates cell function. Age related decline in AMPK plays a key role in the observed loss of function, disordered bioenergetics and metabolism observed in ageing cells and likely contributes to age related disease [91]. Indeed OA chondrocytes are deficient in the metabolic biosensors active AMPK [84].

In our previous cartilage ageing equine transcriptomics study [22] we identified an over representation of DEGs (principally reduced in ageing) involved in pathways for extracellular matrix, degradative proteases and matrix synthetic enzymes and cytokines/growth factors and Wnt signalling. In our aged tissue engineered cartilage DEGs principle pathways involved mitochondrial dysfunction and oxidative phosphorylation. In both ageing cartilage and ageing tissue engineered cartilage the pathway hepatic fibrosis was identified with the genes COL2, COL9 and COL24 DE in both data sets. However for cartilage this was evident as reduced expression in ageing whereas in tissue-engineered cartilage they were increase in ageing. Thus the age-related changes in cartilage are not reflected using tissue-engineered cartilage. Our Achilles tendon ageing RNASeq study [23] determined the top networks for DEGs were from cellular function, cellular growth, and cellular cycling pathways. In tissue-engineered tendon the main pathways from DEGs were signalling pathways with large proportion of DEGs being transcription factors. There were no overlaps in the pathways with ageing between tendon and tissue-engineered tendon. It seems that from these results that age-related changes in cartilage and tendon are not reflected at the transcriptome level to those in MSC-derived tissue engineered cartilage and tendon. This could be due to the method/conditions of tissue generation, a more embryonic phenotype in engineered tissues [92](which may be altered with the addition of loading or growth factors [93]) or disparate ageing mechanism’s. Thus whilst tissue-engineering may provide too artificial a systems to study age-related changes in MSC biology per-se, our findings are important in relation to therapeutic cell source decisions.

In tissues derived from ageing MSCs we identified changes in expression level and differences in the relative balance of splice products. AS; the production of multiple mRNA isoforms from a single gene due to alternative choice of exons or splice sites during pre-mRNA splicing is a major source of protein diversity for higher organisms, and is frequently regulated in a tissue-specific manner. It is estimated that up to 90 percent of human genes have multiple isoforms [94]. Splice variants from the same gene can produce proteins with discrete properties and diverse (including antagonistic) functions. Furthermore, a number of genetic mutations involved in human disease have been mapped to changes in splicing signals or sequences that regulate splicing. Thus, an understanding of changes in splicing patterns is critical to a comprehensive understanding of biological regulation and disease mechanisms. There is a growing interest in the role of AS in normal tissues [95], development [96] and disease (reviewed [97]), but little is known on its role in MSC ageing and tissue engineering. Changes in AS may have a major impact on cell survival [98] and post-translational modifications [99].

Our study demonstrated that donor MSC age has an effect on splicing events in all engineered tissue types, similar to an ageing study in peripheral blood leukocytes [100], suggesting that modification of mRNA processing may be a feature of human ageing. GO ontology demonstrated enrichment in genes associated principally with metabolic processes in genes undergoing AS in all engineered tissue types. AS in metabolic processes is a frequent phenomenon in diseases such as cancer [101] but also in ageing brain [102] and MSCs [48]. Further analysis of the tenogenic AS genes identified carbohydrate and lipid metabolism as significant metabolic pathways affected in ageing. In tenogenic tissuesthe principal network identified in IPA was cell signalling, interaction function and maintenance. This suggests that similar to some cancer cells [98] in tendon tissues derived from ageing MSCs there may be expression of isoforms that alter cell survival. We have previously observed an age-associated disruption to the balance of alternatively expressed isoforms for selected genes in tendon ageing [23]. In AS affected genes for chondrogenic tissues with age were related to cell death and survival, and growth and proliferation of connective tissue. Interestingly a recent study found pyruvate dehydrogenase kinase isoform 2-mediated alternative splicing switches hypoxia-inducible death protein from cell death to cell survival in cancer cells [103].

Though there was little overlap between DEG and DML those that were displayed a good correlation of DNA methylation with differentially expressed genes (promoter increased methylation; reduced gene expression, gene body increased methylation; increased gene expression). There are a number of possibilities as to why correlation between gene expression and DML was poor. DNA methylation is stable so the methylation changes evident may be associated with ancestral gene expression differences. For instance in the study we differentiated MSCs for 21–28 days. There may have been a gene expression changes between day 0 and 7 accompanied by a methylation change. The gene expression could then return back to basal day 0 levels at day 28 when gene expression was measured, but the methylation change remains. Thus a DNA methylation change may not be accompanied by gene expression change as although gene expression did alter, it is not different at the time points measured. Another possibility is that gene enhancers can be located within the gene body of a different gene. Thus a DML within the gene body of gene A may actually be within the enhancer of gene B and so the DML may be associated with a change in gene B but not gene A in which we assessed the effect of the DML on gene A. Finally gene body methylation can be associated with alternative splicing and transcription from alternative/cryptic transcription start site. These may not be have been detected in our RNASeq analysis.

DNA methylation provides a stable and heritable gene regulatory mechanism enabling cells to alter the expression of many genes. Alterations in DML have previously been seen in ageing tissues [104] as a global hypomethylation, as well as aged MSCs [48, 49]. Significant age-related DMLs, both tissue specific and common were identified in all tissue types. DNA methylation also has a role in genomic imprinting (the epigenetic occurrence by which certain genes are expressed in a parent-of-origin-given manner) by regulating the differential expression of maternal and paternal imprinted genes. It is also important in DNA damage/repair and genomic instability [105]. Furthermore a number of disease have been associated with aberrant DNA methylation (reviewed [106]). Thus alterations in methylation in engineered tissues may have further consequences than gene expression changes alone. For instance altered methylation may affect the DNA damage response resulting in senescence and apoptosis [107]. Further work is required to decipher the impact of the DNA methylation changes evident in this study on DNA damage and genomic instability in musculoskeletal engineered tissues with ageing.

Methylation has dual and opposing roles in the gene expression regulation. In promoter regions DNA methylation is correlated with transcriptional repression, while in gene bodies it is generally associated with high expression levels [49]. This paradox emphasizes the diverse involvement of methylation in specific genomic and cellular contexts. We described common age-related pathways from DML of skeletal system morphogenesis, regulation of transcription regulation (both principally due to DML in HOX genes) and cell proliferation (also identified in RNASeq data).

The network ‘skeletal and muscular development and function’ significant in all tissues due to DMLs was principally due to the enrichment of homeotic or HOX genes, similar to studies in ageing MSCs [24, 49]. HOX gene expression is tightly regulated temporally during vertebrate development. An association between HOX genes and longevity has been previously proposed [108]. HOXB7 (chondrogenic), HOXB6 and HOXB7 (osteogenic) and HOXA3 and HOXB6 (tenogenic) were also DEGs in the RNASeq study. However, for HOXB7 (osteogenic) and HOXA3 (tenogenic) DEG did not correlate with DML position. Despite this, the dysregulation of HOX genes at the mRNA and epigenetic level consolidate their role in both MSC ageing and in engineered tissues from ageing MSCs.

An interesting signature of age-related DML was that between 40% (chondrogenic and osteogenic) and 50% (tenogenic) of the top 20 most DML for each engineered tissue type were transcription factors. DML not only transcriptionally regulates gene expression but in ageing we identified significant regulation of the transcription factors. In our study of ageing tendon we identified an overrepresentation of DE transcription factors in ageing tendon [23] suggesting they may have a pivotal role in tendon ageing and tissue engineered tendon. Conversely in our cartilage ageing study there were few age-related DE transcription factors. Furthermore many age-related DML identified in all engineered tissue types resulted in ‘differentiation of cells’ being highlighted. Site specific CpG site methylation changes can affect MSC chondrogenic differentiation [109] whilst alterations in MSC potential have been previously noted [110]. However ‘cell survival’ networks relating to DML were affected in chondrogenic and osteogenic tissues only suggesting distinct age-related methylation patterns between tissue types with potential distinct consequences to engineered tissues.

There was an age-related activation in the function differentiation in all tissues due to DMLs. The DM at some of these loci could be responsible for changes in differentiation potential previously described in chondrogenesis and osteogenesis from ageing MSCs. Studies have described a more rapid decline in differentiation potential for osteoblastic and chondrogenic lineages relative to adipogenic differentiation [90, 111]. No studies have investigated tenogenic differentiation potential with age. Our results would suggest based on the amount of gene expression changes, AS and DML alterations that tenogenic differentiation is likely to be more maintained with ageing compared to chondrogenic and osteogenic lineages. However this hypothesis needs validation. Additional parameters such as histone modification would benefit the analysis but this was beyond the financial scope of the study. Dynamic histone methylation can contribute to the ageing process through influencing DNA repair and transcriptional regulation of ageing processes (reviewed [112]).

## Conclusion

Context-dependent DNA methylation plays a critical role in regulating gene transcription, thereby serving as an important epigenetic marker or regulator in many biological activities. Taken together our RNASeq and DNA methylation results in engineered tissues suggest an age-related loss in the differentiated cells ability to respond to biological cues. Age-related cellular dysfunctions have been hypothesized to results in musculoskeletal age-related diseases such as OA, osteoporosis and tendinopathy. These results have significant implications for therapeutic cell source decisions (autologous or allogeneic) revealing the necessity of approaches to improve functionality of ageing MSCs.

## Supporting Information

S1 File Table of primer sequences. (DOCX)

S2 File Supplementary methods. (DOCX)

S3 File RNASeq reads summary table. (XLSX)

S4 File SmallRNASeq reads summary table. (XLS)

S5 File Table of top 10 expressed snoRNAs and microRNAs using FPKM. (XLSX)

S6 File Age-related differentially expressed genes fromtissues (±1.4 log2 fold change, FDR<0.05) for each tissues type. (XLSX)

S7 File RNASeq data including FPKM. (XLSX)

S8 File MA plots RNASeq and smallSeq. (DOCX)

S9 File Tables for each tissue type containing genes in which there was a significant difference in splicing events. (XLSX)

S10 File MicroRNA-mRNA expression pairing using differentially expressed miRs and mRNA. (DOCX)

S11 File Tables for each engineered tissue type of differentially methylated loci (with FDR<0.05 and 10% mean methylation difference). (XLSX)

S12 File Tables for each tissue type of DM at the site, promoter, CgG isalnd and gene level (FDR adjusted p-value <0.05 and methylation difference of 15% for promter CpG island and genes and FDR adjusted p-value <0.01 and methylation difference of 15% at site level). (XLSX)

## References

[1] Orth P, Rey-Rico A, Venkatesan JK, Madry H, Cucchiarini M. Current perspectives in stem cell research for knee cartilage repair. Stem cells and cloning: advances and applications. 2014;7:1–17. 10.2147/SCCAA.S42880 24520197 PMC3897321

[2] Gomez-Barrena E, Rosset P, Lozano D, Stanovici J, Ermthaller C, Gerbhard F. Bone fracture healing: cell therapy in delayed unions and nonunions. Bone. 2015;70:93–101. 10.1016/j.bone.2014.07.033 . 25093266

[3] Docheva D, Muller SA, Majewski M, Evans CH. Biologics for tendon repair. Advanced drug delivery reviews. 2015;84:222–39. 10.1016/j.addr.2014.11.015 25446135 PMC4519231

[4] Oh J, Lee YD, Wagers AJ. Stem cell aging: mechanisms, regulators and therapeutic opportunities. Nature medicine. 2014;20(8):870–80. 10.1038/nm.3651 25100532 PMC4160113

[5] Scharstuhl A, Schewe B, Benz K, Gaissmaier C, Buhring HJ, Stoop R. Chondrogenic potential of human adult mesenchymal stem cells is independent of age or osteoarthritis etiology. Stem Cells. 2007;25(12):3244–51. Epub 2007/09/18. 2007–0300 [pii] 10.1634/stemcells.2007-0300 . 17872501

[6] Li C, Wei G, Gu Q, Wen G, Qi B, Xu L, et al Donor Age and Cell Passage Affect Osteogenic Ability of Rat Bone Marrow Mesenchymal Stem Cells. Cell Biochem Biophys. 2015 Epub 2015/01/31. 10.1007/s12013-014-0500-9 . 25634304

[7] Zaim M, Karaman S, Cetin G, Isik S. Donor age and long-term culture affect differentiation and proliferation of human bone marrow mesenchymal stem cells. Ann Hematol. 2012;91(8):1175–86. Epub 2012/03/08. 10.1007/s00277-012-1438-x . 22395436

[8] Stolzing A, Jones E, McGonagle D, Scutt A. Age-related changes in human bone marrow-derived mesenchymal stem cells: consequences for cell therapies. Mech Ageing Dev. 2008;129(3):163–73. Epub 2008/02/05. 10.1016/j.mad.2007.12.002 S0047-6374(07)00179-0 [pii]. . 18241911

[9] Dexheimer V, Mueller S, Braatz F, Richter W. Reduced reactivation from dormancy but maintained lineage choice of human mesenchymal stem cells with donor age. PLoS One. 2011;6(8):e22980 Epub 2011/08/19. 10.1371/journal.pone.0022980 PONE-D-11-04667 [pii]. 21850247 PMC3151268

[10] Payne KA, Didiano DM, Chu CR. Donor sex and age influence the chondrogenic potential of human femoral bone marrow stem cells. Osteoarthritis Cartilage. 2010;18(5):705–13. Epub 2010/02/23. 10.1016/j.joca.2010.01.011 S1063-4584(10)00041-5 [pii]. 20171308 PMC2862807

[11] Brady K, Dickinson SC, Guillot PV, Polak J, Blom AW, Kafienah W, et al Human fetal and adult bone marrow-derived mesenchymal stem cells use different signaling pathways for the initiation of chondrogenesis. Stem Cells Dev. 2014;23(5):541–54. Epub 2013/11/01. 10.1089/scd.2013.0301 24172175 PMC3929258

[12] Bergman RJ, Gazit D, Kahn AJ, Gruber H, McDougall S, Hahn TJ. Age-related changes in osteogenic stem cells in mice. J Bone Miner Res. 1996;11(5):568–77. Epub 1996/05/01. 10.1002/jbmr.5650110504 . 9157771

[13] Fickert S, Schroter-Bobsin U, Gross AF, Hempel U, Wojciechowski C, Rentsch C, et al Human mesenchymal stem cell proliferation and osteogenic differentiation during long-term ex vivo cultivation is not age dependent. J Bone Miner Metab. 2011;29(2):224–35. Epub 2010/09/03. 10.1007/s00774-010-0215-y . 20811759

[14] Stenderup K, Justesen J, Clausen C, Kassem M. Aging is associated with decreased maximal life span and accelerated senescence of bone marrow stromal cells. Bone. 2003;33(6):919–26. Epub 2003/12/18. S8756328203002679 [pii]. . 14678851 10.1016/j.bone.2003.07.005

[15] Leskela HV, Risteli J, Niskanen S, Koivunen J, Ivaska KK, Lehenkari P. Osteoblast recruitment from stem cells does not decrease by age at late adulthood. Biochem Biophys Res Commun. 2003;311(4):1008–13. Epub 2003/11/19. S0006291X0302179X [pii]. . 14623282 10.1016/j.bbrc.2003.10.095

[16] Alt EU, Senst C, Murthy SN, Slakey DP, Dupin CL, Chaffin AE, et al Aging alters tissue resident mesenchymal stem cell properties. Stem Cell Res. 2012;8(2):215–25. Epub 2012/01/24. 10.1016/j.scr.2011.11.002 S1873-5061(11)00156-5 [pii]. . 22265741

[17] Kohler J, Popov C, Klotz B, Alberton P, Prall WC, Haasters F, et al Uncovering the cellular and molecular changes in tendon stem/progenitor cells attributed to tendon aging and degeneration. Aging Cell. 2013;12(6):988–99. Epub 2013/07/06. 10.1111/acel.12124 23826660 PMC4225469

[18] Chen L, Wang GD, Liu JP, Wang HS, Liu XM, Wang Q, et al miR-135a modulates tendon stem/progenitor cell senescence via suppressing ROCK1. Bone. 2015;71:210–6. Epub 2014/12/03. 10.1016/j.bone.2014.11.001 S8756-3282(14)00400-1 [pii]. . 25460182

[19] Blakeley P, Fogarty NM, Del Valle I, Wamaitha SE, Hu TX, Elder K, et al Defining the three cell lineages of the human blastocyst by single-cell RNA-seq. Development. 2015;142(20):3613 Epub 2015/10/22. 10.1242/dev.131235 142/20/3613 [pii]. 26487783 PMC4631772

[20] Giorda K, Sun Y, Frey E, Taylor M, Barron T, Piper D, et al RNA-seq to identify novel markers for neural tissue differentiation. The Journal of the Federation of American Societies for Experimental Biology. 2014;28(1 (Supplement)):LB211.

[21] Rushton MD, Reynard LN, Barter MJ, Refaie R, Rankin KS, Young DA, et al Characterization of the cartilage DNA methylome in knee and hip osteoarthritis. Arthritis Rheumatol. 2014;66(9):2450–60. Epub 2014/05/20. 10.1002/art.38713 24838673 PMC4314681

[22] Peffers M, Liu X, Clegg P. Transcriptomic signatures in cartilage ageing. Arthritis research & therapy. 2013;15(4):R98 10.1186/ar4278 23971731 PMC3978620

[23] Peffers MJ, Fang Y, Cheung K, Wei TK, Clegg PD, Birch HL. Transcriptome analysis of ageing in uninjured human Achilles tendon. Arthritis research & therapy. 2015;17:33 10.1186/s13075-015-0544-2 25888722 PMC4355574

[24] Peffers MJ, Collins J, Fang Y, Goljanek-Whysall K, Rushton M, Loughlin J, et al Age-related changes in mesenchymal stem cells identified using a multi-omics approach. Eur Cell Mater. 2016;31:136–59. Epub 2016/02/09. vol031a10 [pii]. . 26853623 10.22203/ecm.v031a10

[25] Kanawa M, Igarashi A, Ronald VS, Higashi Y, Kurihara H, Sugiyama M, et al Age-dependent decrease in the chondrogenic potential of human bone marrow mesenchymal stromal cells expanded with fibroblast growth factor-2. Cytotherapy. 2013;15(9):1062–72. 10.1016/j.jcyt.2013.03.015 . 23800732

[26] Sun HJ, Bahk YY, Choi YR, Shim JH, Han SH, Lee JW. A proteomic analysis during serial subculture and osteogenic differentiation of human mesenchymal stem cell. Journal of orthopaedic research: official publication of the Orthopaedic Research Society. 2006;24(11):2059–71. 10.1002/jor.20273 . 16947300

[27] Kapacee Z, Yeung CY, Lu Y, Crabtree D, Holmes DF, Kadler KE. Synthesis of embryonic tendon-like tissue by human marrow stromal/mesenchymal stem cells requires a three-dimensional environment and transforming growth factor beta3. Matrix biology: journal of the International Society for Matrix Biology. 2010;29(8):668–77. 10.1016/j.matbio.2010.08.005 20736064 PMC3611595

[28] Peffers MJ, Thornton DJ, Clegg PD. Characterization of neopeptides in equine articular cartilage degradation. Journal of orthopaedic research: official publication of the Orthopaedic Research Society. 2016;34(1):106–20. 10.1002/jor.22963 26124002 PMC4737130

[29] Paul H, Reginato AJ, Schumacher HR. Alizarin red S staining as a screening test to detect calcium compounds in synovial fluid. Arthritis and rheumatism. 1983;26(2):191–200. . 6186260 10.1002/art.1780260211

[30] Bancroft JD, Gamble M, editors. heory and practice of histological techniques: Elsevier Health Sciences; 2008.

[31] Chomczynski P, Sacchi N. Single-step method of RNA isolation by acid guanidinium thiocyanate-phenol-chloroform extraction. Anal Biochem. 1987;162(1):156–9. Epub 1987/04/01. 0003-2697(87)90021-2 [pii]. . 2440339 10.1006/abio.1987.9999

[32] Benjamini Y, Hochberg Y. Controlling the false discovery rate: a practical and powerful approach to multiple testing. Methodology. 1995;57(1):289–300.

[33] Pidsley R, CC YW, Volta M, Lunnon K, Mill J, Schalkwyk LC. A data-driven approach to preprocessing Illumina 450K methylation array data. BMC genomics. 2013;14:293 10.1186/1471-2164-14-293 23631413 PMC3769145

[34] Whitaker JW, Shoemaker R, Boyle DL, Hillman J, Anderson D, Wang W, et al An imprinted rheumatoid arthritis methylome signature reflects pathogenic phenotype. Genome medicine. 2013;5(4):40 10.1186/gm444 23631487 PMC3706831

[35] Mi H, Muruganujan A, Thomas PD. PANTHER in 2013: modeling the evolution of gene function, and other gene attributes, in the context of phylogenetic trees. Nucleic Acids Res. 2013;41(Database issue):D377–86. Epub 2012/11/30. 10.1093/nar/gks1118 gks1118 [pii]. 23193289 PMC3531194

[36] Huang DW, Sherman BT, Lempicki RA. Systematic and integrative analysis of large gene lists using DAVID bioinformatics resources. Nat Protoc. 2009;4(1):44–57. 10.1038/nprot.2008.211. ISI:000265781800006. 19131956

[37] URL3. Ingenuity systems. Ingenuity pathway analysis [http://www.ingenuity.com/]

[38] http://www.targetscan.org/.

[39] Martin I, Jakob M, Schafer D, Dick W, Spagnoli G, Heberer M. Quantitative analysis of gene expression in human articular cartilage from normal and osteoarthritic joints. Osteoarthritis Cartilage. 2001;9(2):112–8. Epub 2001/03/10. 10.1053/joca.2000.0366 S1063-4584(00)90366-2 [pii]. . 11237658

[40] Livak KJ, Schmittgen TD. Analysis of relative gene expression data using real-time quantitative PCR and the 2(-Delta Delta C(T)) Method. Methods. 2001;25(4):402–8. Epub 2002/02/16. 10.1006/meth.2001.1262 S1046-2023(01)91262-9 [pii]. . 11846609

[41] Mueller AM. A Systems Biology Approach To Musculoskeletal Tissue Engineering: Transcriptomic And Proteomic Analysis Of Cartilage And Tendon Cells. Liverpool: University of Liverpool; 2015.

[42] ftp://ftp.ensembl.org/pub/release-73/gtf/homo_sapiens/Homo_sapiens.GRCh37.73.gtf.gz 2015.

[43] O'Loughlin A, Lynn DJ, McGee M, Doyle S, McCabe M, Earley B. Transcriptomic analysis of the stress response to weaning at housing in bovine leukocytes using RNA-seq technology. BMC Genomics. 2012;13:250 10.1186/1471-2164-13-250 22708644 PMC3583219

[44] Buitrago DH, Patnaik SK, Kadota K, Kannisto E, Jones DR, Adusumilli PS. Small RNA sequencing for profiling microRNAs in long-term preserved formalin-fixed and paraffin-embedded non-small cell lung cancer tumor specimens. PLoS One. 2015;10(3):e0121521 Epub 2015/03/27. 10.1371/journal.pone.0121521 PONE-D-14-37715 [pii]. 25812157 PMC4374839

[45] Yang KC, Yamada KA, Patel AY, Topkara VK, George I, Cheema FH, et al Deep RNA sequencing reveals dynamic regulation of myocardial noncoding RNAs in failing human heart and remodeling with mechanical circulatory support. Circulation. 2014;129(9):1009–21. Epub 2014/01/17. 10.1161/CIRCULATIONAHA.113.003863 CIRCULATIONAHA.113.003863 [pii]. 24429688 PMC3967509

[46] Jjingo D, Conley AB, Yi SV, Lunyak VV, Jordan IK. On the presence and role of human gene-body DNA methylation. Oncotarget. 2012;3(4):462–74. Epub 2012/05/12. 497 [pii]. 22577155 10.18632/oncotarget.497 PMC3380580

[47] Natsu K, Ochi M, Mochizuki Y, Hachisuka H, Yanada S, Yasunaga Y. Allogeneic bone marrow-derived mesenchymal stromal cells promote the regeneration of injured skeletal muscle without differentiation into myofibers. Tissue engineering. 2004;10(7–8):1093–112. 10.1089/ten.2004.10.1093 . 15363167

[48] Peffers MJ, Collins J, Fang Y, Goljanek-Whysall K, Rushton M, Loughlin J, et al Age-related changes in mesenchymal stem cells identified using a multi-omics approach. European Cells and Materials. 2016;accepted. 10.22203/ecm.v031a10 26853623

[49] Bork S, Pfister S, Witt H, Horn P, Korn B, Ho AD, et al DNA methylation pattern changes upon long-term culture and aging of human mesenchymal stromal cells. Aging Cell. 2010;9(1):54–63. Epub 2009/11/10. 10.1111/j.1474-9726.2009.00535.x ACE535 [pii]. 19895632 PMC2814091

[50] Knuth CA, Clark ME, Meeson AP, Khan SK, Dowen DJ, Deehan DJ, et al Low oxygen tension is critical for the culture of human mesenchymal stem cells with strong osteogenic potential from haemarthrosis fluid. Stem Cell Rev. 2013;9(5):599–608. Epub 2013/06/14. 10.1007/s12015-013-9446-3 . 23760649

[51] Delgado-Calle J, Riancho JA. The role of DNA methylation in common skeletal disorders. Biology (Basel). 2012;1(3):698–713. Epub 2012/01/01. 10.3390/biology1030698 biology1030698 [pii]. 24832515 PMC4009801

[52] Wei J, Li H, Wang S, Li T, Fan J, Liang X, et al let-7 enhances osteogenesis and bone formation while repressing adipogenesis of human stromal/mesenchymal stem cells by regulating HMGA2. Stem Cells Dev. 2014;23(13):1452–63. 10.1089/scd.2013.0600 24617339 PMC4066225

[53] Wu T, Liu Y, Fan Z, Xu J, Jin L, Gao Z, et al miR-21 Modulates the Immunoregulatory Function of Bone Marrow Mesenchymal Stem Cells Through the PTEN/Akt/TGF-beta1 Pathway. Stem Cells. 2015;33(11):3281–90. 10.1002/stem.2081 . 26086742

[54] Ding W, Li J, Singh J, Alif R, Vazquez-Padron RI, Gomes SA, et al miR-30e targets IGF2-regulated osteogenesis in bone marrow-derived mesenchymal stem cells, aortic smooth muscle cells, and ApoE-/- mice. Cardiovascular research. 2015;106(1):131–42. 10.1093/cvr/cvv030 25678587 PMC4375408

[55] Laine SK, Alm JJ, Virtanen SP, Aro HT, Laitala-Leinonen TK. MicroRNAs miR-96, miR-124, and miR-199a regulate gene expression in human bone marrow-derived mesenchymal stem cells. J Cell Biochem. 2012;113(8):2687–95. 10.1002/jcb.24144 . 22441842

[56] Wang JM, Tao J, Chen DD, Cai JJ, Irani K, Wang Q, et al MicroRNA miR-27b rescues bone marrow-derived angiogenic cell function and accelerates wound healing in type 2 diabetes mellitus. Arteriosclerosis, thrombosis, and vascular biology. 2014;34(1):99–109. 10.1161/ATVBAHA.113.302104 . 24177325 PMC5533613

[57] Nakamura Y, Inloes JB, Katagiri T, Kobayashi T. Chondrocyte-specific microRNA-140 regulates endochondral bone development and targets Dnpep to modulate bone morphogenetic protein signaling. Mol Cell Biol. 2011;31(14):3019–28. 10.1128/MCB.05178-11 21576357 PMC3133397

[58] Pivetta E, Scapolan M, Pecolo M, Wassermann B, Abu-Rumeileh I, Balestreri L, et al MMP-13 stimulates osteoclast differentiation and activation in tumour breast bone metastases. Breast cancer research: BCR. 2011;13(5):R105 10.1186/bcr3047 22032644 PMC3262218

[59] Le LT, Swingler TE, Crowe N, Vincent TL, Barter MJ, Donell ST, et al The microRNA-29 family in cartilage homeostasis and osteoarthritis. Journal of molecular medicine. 2015 10.1007/s00109-015-1374-z . 26687115 PMC4856728

[60] Kawase Y, Hoshino T, Yokota K, Kuzuhara A, Nakamura M, Maeda Y, et al Bone malformations in interleukin-18 transgenic mice. J Bone Miner Res. 2003;18(6):975–83. 10.1359/jbmr.2003.18.6.975 . 12817749

[61] Wang H, Beyer I, Persson J, Song H, Li Z, Richter M, et al A new human DSG2-transgenic mouse model for studying the tropism and pathology of human adenoviruses. Journal of virology. 2012;86(11):6286–302. 10.1128/JVI.00205-12 22457526 PMC3372198

[62] Zhao Y, Wu D, Fei C, Guo J, Gu S, Zhu Y, et al Down-regulation of Dicer1 promotes cellular senescence and decreases the differentiation and stem cell-supporting capacities of mesenchymal stromal cells in patients with myelodysplastic syndrome. Haematologica. 2015;100(2):194–204. 10.3324/haematol.2014.109769 25361944 PMC4803146

[63] Tan J, Xu X, Tong Z, Lin J, Yu Q, Lin Y, et al Decreased osteogenesis of adult mesenchymal stem cells by reactive oxygen species under cyclic stretch: a possible mechanism of age related osteoporosis. Bone research. 2015;3:15003 10.1038/boneres.2015.3 26273536 PMC4413016

[64] Millar NL, Gilchrist DS, Akbar M, Reilly JH, Kerr SC, Campbell AL, et al MicroRNA29a regulates IL-33-mediated tissue remodelling in tendon disease. Nature communications. 2015;6:6774 10.1038/ncomms7774 25857925 PMC4403384

[65] Mendias CL, Gumucio JP, Lynch EB. Mechanical loading and TGF-beta change the expression of multiple miRNAs in tendon fibroblasts. Journal of applied physiology. 2012;113(1):56–62. 10.1152/japplphysiol.00301.2012 22539168 PMC3404830

[66] Zhang YP, Peng XY, Li ZH, Chen FH. Hyperglycemic effects of a periocular dexamethasone injection in diabetic patients after vitreoretinal surgery. Biomedical and environmental sciences: BES. 2012;25(3):311–6. 10.3967/0895-3988.2012.03.009 . 22840582

[67] Grammatikakis I, Panda AC, Abdelmohsen K, Gorospe M. Long noncoding RNAs (lncRNAs) and the molecular hallmarks of aging. Ageing 2014;6(12):992–1009. 10.18632/aging.100710 PMC4298369 25543668

[68] Mercer TR, Dinger ME, Mattick JS. Long non-coding RNAs: insights into functions. Nat Rev Genet. 2009;10(3):155–9. Epub 2009/02/04. 10.1038/nrg2521 nrg2521 [pii]. . 19188922

[69] Abdelmohsen K, Panda A, Kang MJ, Xu J, Selimyan R, Yoon JH, et al Senescence-associated lncRNAs: senescence-associated long noncoding RNAs. Aging Cell. 2013;12(5):890–900. Epub 2013/06/14. 10.1111/acel.12115 23758631 PMC3773026

[70] Aloe L, Tuveri MA, Carcassi U, Levi-Montalcini R. Nerve growth factor in the synovial fluid of patients with chronic arthritis. Arthritis and rheumatism. 1992;35(3):351–5. Epub 1992/03/01. . 1536673 10.1002/art.1780350315

[71] Nockher WA, Renz H. Neurotrophins in clinical diagnostics: pathophysiology and laboratory investigation. Clin Chim Acta. 2005;352(1–2):49–74. Epub 2005/01/18. S0009-8981(04)00478-4 [pii] 10.1016/j.cccn.2004.10.002 . 15653100

[72] Iannone F, De Bari C, Dell'Accio F, Covelli M, Patella V, Lo Bianco G, et al Increased expression of nerve growth factor (NGF) and high affinity NGF receptor (p140 TrkA) in human osteoarthritic chondrocytes. Rheumatology (Oxford). 2002;41(12):1413–8. Epub 2002/12/07. . 12468822 10.1093/rheumatology/41.12.1413

[73] Grimsholm O, Guo Y, Ny T, Forsgren S. Expression patterns of neurotrophins and neurotrophin receptors in articular chondrocytes and inflammatory infiltrates in knee joint arthritis. Cells Tissues Organs. 2008;188(3):299–309. Epub 2008/03/20. 10.1159/000121432 000121432 [pii]. . 18349525

[74] Katoh-Semba R, Semba R, Takeuchi IK, Kato K. Age-related changes in levels of brain-derived neurotrophic factor in selected brain regions of rats, normal mice and senescence-accelerated mice: a comparison to those of nerve growth factor and neurotrophin-3. Neurosci Res. 1998;31(3):227–34. Epub 1998/11/11. S0168-0102(98)00040-6 [pii]. . 9809668 10.1016/s0168-0102(98)00040-6

[75] Stelnicki EJ, Komuves LG, Kwong AO, Holmes D, Klein P, Rozenfeld S, et al HOX homeobox genes exhibit spatial and temporal changes in expression during human skin development. The Journal of investigative dermatology. 1998;110(2):110–5. 10.1046/j.1523-1747.1998.00092.x . 9457903

[76] Ackema KB, Charite J. Mesenchymal stem cells from different organs are characterized by distinct topographic Hox codes. Stem Cells Dev. 2008;17(5):979–91. Epub 2008/06/07. 10.1089/scd.2007.0220 . 18533811

[77] Chang JW, Tsai HL, Chen CW, Yang HW, Yang AH, Yang LY, et al Conditioned mesenchymal stem cells attenuate progression of chronic kidney disease through inhibition of epithelial-to-mesenchymal transition and immune modulation. Journal of cellular and molecular medicine. 2012;16(12):2935–49. 10.1111/j.1582-4934.2012.01610.x 22862802 PMC4393722

[78] Poulsen RC, Knowles HJ, Carr AJ, Hulley PA. Cell differentiation versus cell death: extracellular glucose is a key determinant of cell fate following oxidative stress exposure. Cell Death Dis. 2014;5:e1074 Epub 2014/02/22. 10.1038/cddis.2014.52 cddis201452 [pii]. 24556689 PMC3944267

[79] Collins JA, Wood ST, Nelson KJ, Rowe MA, Carlson CS, Chubinskaya S, et al Oxidative Stress Promotes Peroxiredoxin Hyperoxidation and Attenuates Pro-survival Signalling in Aging Chondrocytes. J Biol Chem. 2016 Epub 2016/01/23. jbc.M115.693523 [pii]M115.693523 [pii] 10.1074/jbc.M115.693523 . 26797130 PMC4807251

[80] Chan DC. Mitochondria: dynamic organelles in disease, aging, and development. Cell. 2006;125(7):1241–52. 10.1016/j.cell.2006.06.010 . 16814712

[81] Loeser RF, Gandhi U, Long DL, Yin W, Chubinskaya S. Aging and oxidative stress reduce the response of human articular chondrocytes to insulin-like growth factor 1 and osteogenic protein 1. Arthritis Rheumatol. 2014;66(8):2201–9. Epub 2014/03/26. 10.1002/art.38641 24664641 PMC4116467

[82] Henrotin YE, Bruckner P, Pujol JP. The role of reactive oxygen species in homeostasis and degradation of cartilage. Osteoarthritis Cartilage. 2003;11(10):747–55. Epub 2003/09/18. S106345840300150X [pii]. . 13129694 10.1016/s1063-4584(03)00150-x

[83] Guan Y, Zhang Y, Breyer MD. The Role of PPARs in the Transcriptional Control of Cellular Processes. Drug News Perspect. 2002;15(3):147–54. Epub 2003/04/05. 294 [pii]. . 12677257 10.1358/dnp.2002.15.3.840011

[84] Wang Y, Zhao X, Lotz M, Terkeltaub R, Liu-Bryan R. Mitochondrial biogenesis is impaired in osteoarthritis chondrocytes but reversible via peroxisome proliferator-activated receptor gamma coactivator 1alpha. Arthritis Rheumatol. 2015;67(8):2141–53. Epub 2015/05/06. 10.1002/art.39182 25940958 PMC4519411

[85] Haussler MR, Whitfield GK, Haussler CA, Hsieh JC, Thompson PD, Selznick SH, et al The nuclear vitamin D receptor: biological and molecular regulatory properties revealed. J Bone Miner Res. 1998;13(3):325–49. Epub 1998/04/03. 10.1359/jbmr.1998.13.3.325 . 9525333

[86] Goltzman D. Discoveries, drugs and skeletal disorders. Nat Rev Drug Discov. 2002;1(10):784–96. Epub 2002/10/03. 10.1038/nrd916 nrd916 [pii]. . 12360256

[87] Horst RL, Goff JP, Reinhardt TA. Advancing age results in reduction of intestinal and bone 1,25-dihydroxyvitamin D receptor. Endocrinology. 1990;126(2):1053–7. Epub 1990/02/01. 10.1210/endo-126-2-1053 . 2153518

[88] Duque G, El Abdaimi K, Macoritto M, Miller MM, Kremer R. Estrogens (E2) regulate expression and response of 1,25-dihydroxyvitamin D3 receptors in bone cells: changes with aging and hormone deprivation. Biochem Biophys Res Commun. 2002;299(3):446–54. Epub 2002/11/26. S0006291X02026578 [pii]. . 12445821 10.1016/s0006-291x(02)02657-8

[89] Gerstenfeld LC, Zurakowski D, Schaffer JL, Nichols DP, Toma CD, Broess M, et al Variable hormone responsiveness of osteoblast populations isolated at different stages of embryogenesis and its relationship to the osteogenic lineage. Endocrinology. 1996;137(9):3957–68. Epub 1996/09/01. 10.1210/endo.137.9.8756572 . 8756572

[90] Kretlow JD, Jin YQ, Liu W, Zhang WJ, Hong TH, Zhou G, et al Donor age and cell passage affects differentiation potential of murine bone marrow-derived stem cells. BMC Cell Biol. 2008;9:60 Epub 2008/10/30. 10.1186/1471-2121-9-60 1471-2121-9-60 [pii]. 18957087 PMC2584028

[91] Liu-Bryan R, Terkeltaub R. Emerging regulators of the inflammatory process in osteoarthritis. Nat Rev Rheumatol. 2015;11(1):35–44. Epub 2014/10/01. 10.1038/nrrheum.2014.162 nrrheum.2014.162 [pii]. 25266449 PMC4374654

[92] Kharaz YA, Tew SR, Peffers M, Canty-Laird EG, Comerford E. Proteomic differences between native and tissue-engineered tendon and ligament. Proteomics. 2016;16(10):1547–56. 10.1002/pmic.201500459 . 27080496 PMC5132062

[93] Paxton JZ, Hagerty P, Andrick JJ, Baar K. Optimizing an intermittent stretch paradigm using ERK1/2 phosphorylation results in increased collagen synthesis in engineered ligaments. Tissue Eng Part A. 2012;18(3–4):277–84. 10.1089/ten.TEA.2011.0336 21902469 PMC3267962

[94] Wang ET, Sandberg R, Luo S, Khrebtukova I, Zhang L, Mayr C, et al Alternative isoform regulation in human tissue transcriptomes. Nature. 2008;456(7221):470–6. Epub 2008/11/04. 10.1038/nature07509 nature07509 [pii]. 18978772 PMC2593745

[95] Shargunov AV, Krasnov GS, Ponomarenko EA, Lisitsa AV, Shurdov MA, Zverev VV, et al Tissue-specific alternative splicing analysis reveals the diversity of chromosome 18 transcriptome. J Proteome Res. 2014;13(1):173–82. 10.1021/pr400808u . 24320163

[96] McAlinden A. Alternative splicing of type II procollagen: IIB or not IIB? Connect Tissue Res. 2014;55(3):165–76. 10.3109/03008207.2014.908860 24669942 PMC4317353

[97] Tazi J, Bakkour N, Stamm S. Alternative splicing and disease. Biochim Biophys Acta. 2009;1792(1):14–26. 10.1016/j.bbadis.2008.09.017 . 18992329 PMC5632948

[98] Prinos P, Garneau D, Lucier JF, Gendron D, Couture S, Boivin M, et al Alternative splicing of SYK regulates mitosis and cell survival. Nat Struct Mol Biol. 2011;18(6):673–9. 10.1038/nsmb.2040 . 21552259

[99] Sheng JJ, Jin JP. Gene regulation, alternative splicing, and posttranslational modification of troponin subunits in cardiac development and adaptation: a focused review. Front Physiol. 2014;5:165 10.3389/fphys.2014.00165 24817852 PMC4012202

[100] Harries LW, Hernandez D, Henley W, Wood AR, Holly AC, Bradley-Smith RM, et al Human aging is characterized by focused changes in gene expression and deregulation of alternative splicing. Aging Cell. 2011;10(5):868–78. 10.1111/j.1474-9726.2011.00726.x 21668623 PMC3173580

[101] David CJ, Manley JL. Alternative pre-mRNA splicing regulation in cancer: pathways and programs unhinged. Genes Dev. 2010;24(21):2343–64. 10.1101/gad.1973010 21041405 PMC2964746

[102] Tollervey JR, Wang Z, Hortobagyi T, Witten JT, Zarnack K, Kayikci M, et al Analysis of alternative splicing associated with aging and neurodegeneration in the human brain. Genome Res. 2011;21(10):1572–82. 10.1101/gr.122226.111 21846794 PMC3202275

[103] Gang H, Dhingra R, Lin J, Hai Y, Aviv Y, Margulets V, et al PDK2-mediated alternative splicing switches Bnip3 from cell death to cell survival. J Cell Biol. 2015;210(7):1101–15. 10.1083/jcb.201504047 26416963 PMC4586742

[104] Calvanese V, Lara E, Kahn A, Fraga MF. The role of epigenetics in aging and age-related diseases. Ageing research reviews. 2009;8(4):268–76. 10.1016/j.arr.2009.03.004 . 19716530

[105] Robertson KD, Jones PA. DNA methylation: past, present and future directions. Carcinogenesis. 2000;21(3):461–7. . 10688866 10.1093/carcin/21.3.461

[106] Robertson KD. DNA methylation and human disease. Nat Rev Genet. 2005;6(8):597–610. 10.1038/nrg1655 . 16136652

[107] Hanzelmann S, Beier F, Gusmao EG, Koch CM, Hummel S, Charapitsa I, et al Replicative senescence is associated with nuclear reorganization and with DNA methylation at specific transcription factor binding sites. Clin Epigenetics. 2015;7(1):19 10.1186/s13148-015-0057-5 25763115 PMC4356053

[108] Venkataraman K, Futerman AH. Do longevity assurance genes containing Hox domains regulate cell development via ceramide synthesis? FEBS Lett. 2002;528(1–3):3–4. Epub 2002/09/26. S0014579302032489 [pii]. . 12297269 10.1016/s0014-5793(02)03248-9

[109] Zimmermann P, Boeuf S, Dickhut A, Boehmer S, Olek S, Richter W. Correlation of COL10A1 induction during chondrogenesis of mesenchymal stem cells with demethylation of two CpG sites in the COL10A1 promoter. Arthritis and rheumatism. 2008;58(9):2743–53. Epub 2008/09/02. 10.1002/art.23736 . 18759285

[110] Sethe S, Scutt A, Stolzing A. Aging of mesenchymal stem cells. Ageing research reviews. 2006;5(1):91–116. 10.1016/j.arr.2005.10.001 . 16310414

[111] Roura S, Farre J, Soler-Botija C, Llach A, Hove-Madsen L, Cairo JJ, et al Effect of aging on the pluripotential capacity of human CD105+ mesenchymal stem cells. Eur J Heart Fail. 2006;8(6):555–63. Epub 2006/03/02. S1388-9842(05)00330-2 [pii] 10.1016/j.ejheart.2005.11.006 . 16507351

[112] McCauley BS, Dang W. Histone methylation and aging: lessons learned from model systems. Biochim Biophys Acta. 2014;1839(12):1454–62. 10.1016/j.bbagrm.2014.05.008 24859460 PMC4240748

