# Supplementary material for: Decoding the Regulatory Landscape of Ageing in Musculoskeletal Engineered Tissues Using Genome-Wide DNA Methylation and RNASeq
Source: PLoS One. 2016 Aug 17;11(8):e0160517. doi: 10.1371/journal.pone.0160517 (PMC4988628; doi:10.1371/journal.pone.0160517)
Supplement: S1 File — (DOCX) [file pone.0160517.s001.docx]

Supplementary File 1- Primer Sequences

| **Gene** | **Ensemble Gene ID** | **Primer Sequences** |
| --- | --- | --- |
| ACAN^a^ | ENSG00000157766 | F: TCGAGGACAGCGAGGCC |
|  |  | R: TCGAGGGTGTAGCGTGTAGAGA |
| ALX1 | ENSG00000180318 | F: TTTTCACTGACTCTCTTCTTACTGG |
|  |  | R: ATATTGGCGGTGTGCTCCTT |
| COL2A1^a^ | ENSG00000139219 | F: GGCAATAGCAGGTTCACGTACA |
|  |  | R: CGATAACAGTCTTGCCCCACTT |
| HOXB6 | ENSG00000108511 | F: CGCCTCCCCTCCCAATG |
|  |  | R: CGCATAGCCCGACGAATAG |
| HOXB7 | ENSG00000260027 | F: GTTGCCTGCCCTCCTGAG |
|  |  | R: GCGAGTGGTAGGTTTTGGG |
| MAB21L2 | ENSG00000181541 | F: CCGCTCAACAACTACCACAT |
|  |  | R: TCGTCCCAGTCCGTTTCTC |
| MMP16^b^ | ENSG00000156103 | F: ACCCGTGTAACCCTTTGAGA |
|  |  | R: AACCTGAACTTCTTGAACTTGTG |
| RPS8 | ENSG00000142937 | F: CAAGAAATACCGTGCCCTGAG |
|  |  | R: CGATGATCCTTGTTTTACGAGTAC |
| PITX2 | ENSG00000164093 | F: AACTCCGCCCTTGAAAGACT |
|  |  | R: GATTCAGTGGTTTCCTTTTCTCTATA |
| TGFA | ENSG00000163235 | F: GCCACCTCCCCACAACAG |
|  |  | R: ATTAAACAGTTTCCCCTCCTTCAC |

^a^ [[1](#_ENREF_1)],  ^b^ [[2](#_ENREF_2)] denote primer pairs published previously. F, forward; R, reverse. ACAN; aggrecan, ALX1; ALX homeobox 1, COL2A1; collagen II alpha 1, HOXB6; homeobox B6, HOXB7; homeobox B7, MAB21L2; mab-21-like 1 , MMP16; matrix metalloproteinase 16, , RPS8; ribosomal protein 8, PITX2; paired-like homeodomain 2, TGFA; transforming growth factor alpha,

1. Martin, I., et al., *Quantitative analysis of gene expression in human articular cartilage from normal and osteoarthritic joints.* Osteoarthritis Cartilage, 2001. **9**(2): p. 112-8.

2. Peffers, M.J., et al., *Transcriptome analysis of ageing in uninjured human Achilles tendon.* Arthritis Res Ther, 2015. **17**: p. 33.
