# Supplementary material for: Decoding the Regulatory Landscape of Ageing in Musculoskeletal Engineered Tissues Using Genome-Wide DNA Methylation and RNASeq
Source: PLoS One. 2016 Aug 17;11(8):e0160517. doi: 10.1371/journal.pone.0160517 (PMC4988628; doi:10.1371/journal.pone.0160517)
Supplement: S2 File — (DOCX) [file pone.0160517.s002.docx]

Supplementary file 2- Supplementary Methods

***RNA isolation, library preparation for RNASeq and small RNASeq and sequencing***

Total RNA integrity was confirmed using the Agilent 2100 Bioanalyzer (Agilent Technologies, Santa Clara, USA). Ribosomal RNA (rRNA) was depleted from RNA samples by using the Ribo-Zero™ rRNA Removal Kit (Epicentre, Madison, USA) in accordance with the instructions of the manufacturer. For RNASeq cDNA libraries were prepared with the ScriptSeq v2 RNA-Seq Library Preparation Kit (Epicentre, Madison, USA) using 50 ng rRNA depleted RNA as starting material in accordance with manufacturer protocols as previously described [^29^](#_ENREF_29). For both RNASeq and small RNASeq (smallSeq) the quantity and quality of the final pools were assessed using Qubit (Invitrogen™ Life Technologies, USA) and Agilent 2100 Bioanalyzer (Agilent Technologies, Santa Clara, USA). The final pooled library was diluted to 8 pmol before hybridisation. Sequencing was undertaken with the Illumina HiSeq 2000 (Illumina, San Diego, USA) at 2 × 100-base pair (bp) paired-end sequencing with v3 chemistry. 1μg of total RNA were used to prepare small RNA libraries using the NEBNextMultiplex Small RNA Library Preperation Set for Illumina (New England BioLabs, Ipswich, USA) according to manufacturer’s instructions Following 12 cycles of amplification, the PCR amplified cDNA engineered tissues were purified using the Qiagen QIAQuick PCR Purification kit (Qiagen, Crawley, UK). Multiplexed size selected small RNA library pools were sequenced on one lane of the Illumina HiSeq 2500 (Illumina, San Diego, USA) at 1x50 bp sequencing with rapid-run mode chemistry.

***RNA data processing***

The RNASeq sequence libraries were processed by using CASAVA version 1.8.2 to produce 100-bp paired-end sequence data in fastq format. The fastq files were processed, quality trimming performed and the trimmed R1-R2 read pairs were aligned to reference sequence [[1](#_ENREF_1)] as previously described [[2](#_ENREF_2)]. The differential gene expression analysis was undertaken as previously described [[2](#_ENREF_2)]. Differentially expressed genes (DEGs) were extracted by applying the threshold false discovery rate (FDR) of less than 0.05 to adjusted P values, which were generated by using Benjamini and Hochberg approach [[3](#_ENREF_3)].

For analysis of splice variants read counts per gene were calculated using HTSeq-count [[4](#_ENREF_4)]. The gene count data were used to perform a data variation assessment and make a gene level differential expression analysis using edgeR [[5](#_ENREF_5)]. Differential transcript expression and splicing analysis were conducted using Cuffdiff by taking BAM files exported from TopHat as input [[6](#_ENREF_6)].

For smallSeq base-calling and de-multiplexing of indexed reads was performed by CASAVA version 1.8.2 (Illumina, San Diego, USA) to produce data, in fastq format. The raw fastq files were trimmed to remove Illumina adapter sequences using Cutadapt version 1.2.1 [[7](#_ENREF_7)]. The option “-O 3” was set, so the 3' end of any reads which matched the adapter sequence over at least 3 bp was trimmed. The reads were trimmed to remove low quality bases, using Sickle version 1.2 with a minimum window quality score of 20. After trimming, reads shorter than 10 bp were removed.

Trimmed R1 reads were mapped to mature human microRNA (miRNA) and other human small RNAs included in Affymetrix miRNA 4.0 arrays.Reads were mapped to the reference sequences using Bowtie2 version 2.1.0 [[8](#_ENREF_8)]. Single-end mapping was carried out using the “very-sensitive-local” set of options, which increases the sensitivity and accuracy of the alignment relative to the default parameters. In addition, FPKM (fragments per kilobase of exon per million fragments mapped) values were converted from count values for comparing expression levels among small RNAs.

**References**

1. *Human reference genome sequence.* [*ftp://ftp.ensembl.org/pub/release-73/*](ftp://ftp.ensembl.org/pub/release-73/)

*fasta/homo_sapiens/dna/*. 2014.

2. Peffers, M.J., et al., *Transcriptome analysis of ageing in uninjured human Achilles tendon.* Arthritis Res Ther, 2015. **17**: p. 33.

3. Benjamini, Y. and Y. Hochberg, *Controlling the false discovery rate: a practical and powerful approach to multiple testing.* Methodology, 1995. **57**(1): p. 289-300.

4. [*http://www-huber.embl.de/users/anders/HTSeq/doc/count.html*](http://www-huber.embl.de/users/anders/HTSeq/doc/count.html). 2014.

5. Robinson, M.D., D.J. McCarthy, and G.K. Smyth, *edgeR: a Bioconductor package for differential expression analysis of digital gene expression data.* Bioinformatics, 2010. **26**(1): p. 139-40.

6. Trapnell, C., et al., *Differential gene and transcript expression analysis of RNA-seq experiments with TopHat and Cufflinks.* Nat Protoc, 2012. **7**(3): p. 562-78.

7. Martin, M., *Cutadapt removes adapter sequences from highthroughput*

*sequencing reads.* EMBnet.journal 2011. **17**: p. 10-12.

8. Langmead, B. and S.L. Salzberg, *Fast gapped-read alignment with Bowtie 2.* Nat Methods, 2012. **9**(4): p. 357-9.
