# Supplementary material for: Decoding the Regulatory Landscape of Ageing in Musculoskeletal Engineered Tissues Using Genome-Wide DNA Methylation and RNASeq
Source: PLoS One. 2016 Aug 17;11(8):e0160517. doi: 10.1371/journal.pone.0160517 (PMC4988628; doi:10.1371/journal.pone.0160517)

Supplementary File 8- A. MA Plots of differentially expressed RNASeq data analysis of osteogenic, tenogenic and chondrogenic engineered tissues (FDR<0.05 and 1.4 LFC), B. MA Plots of Differentially expressed smallRNASeq data analysis of osteogenic, tenogenic and chondrogenic engineered tissues (FDR<0.05 and 1.4 LFC).

A.


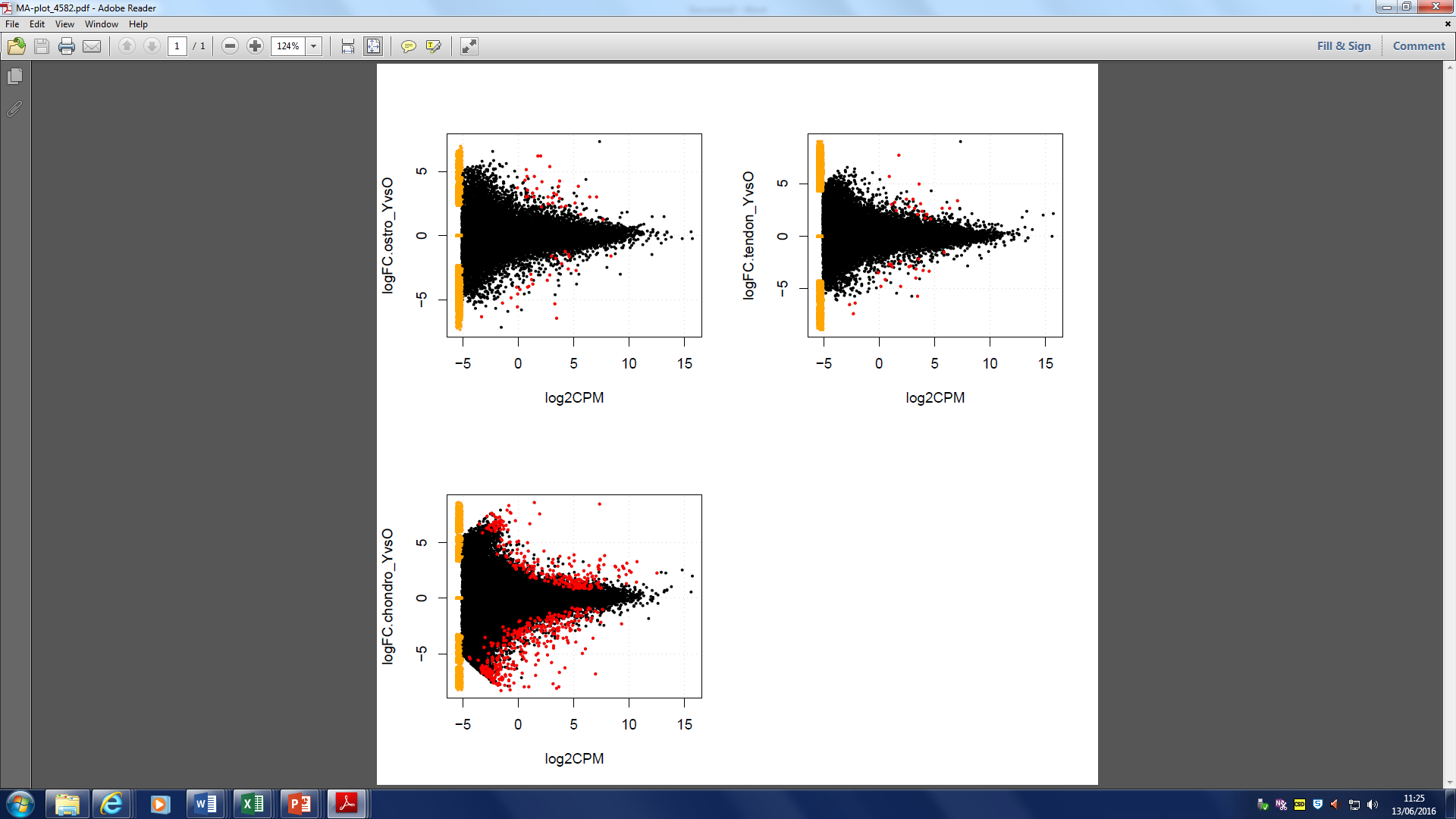


B.


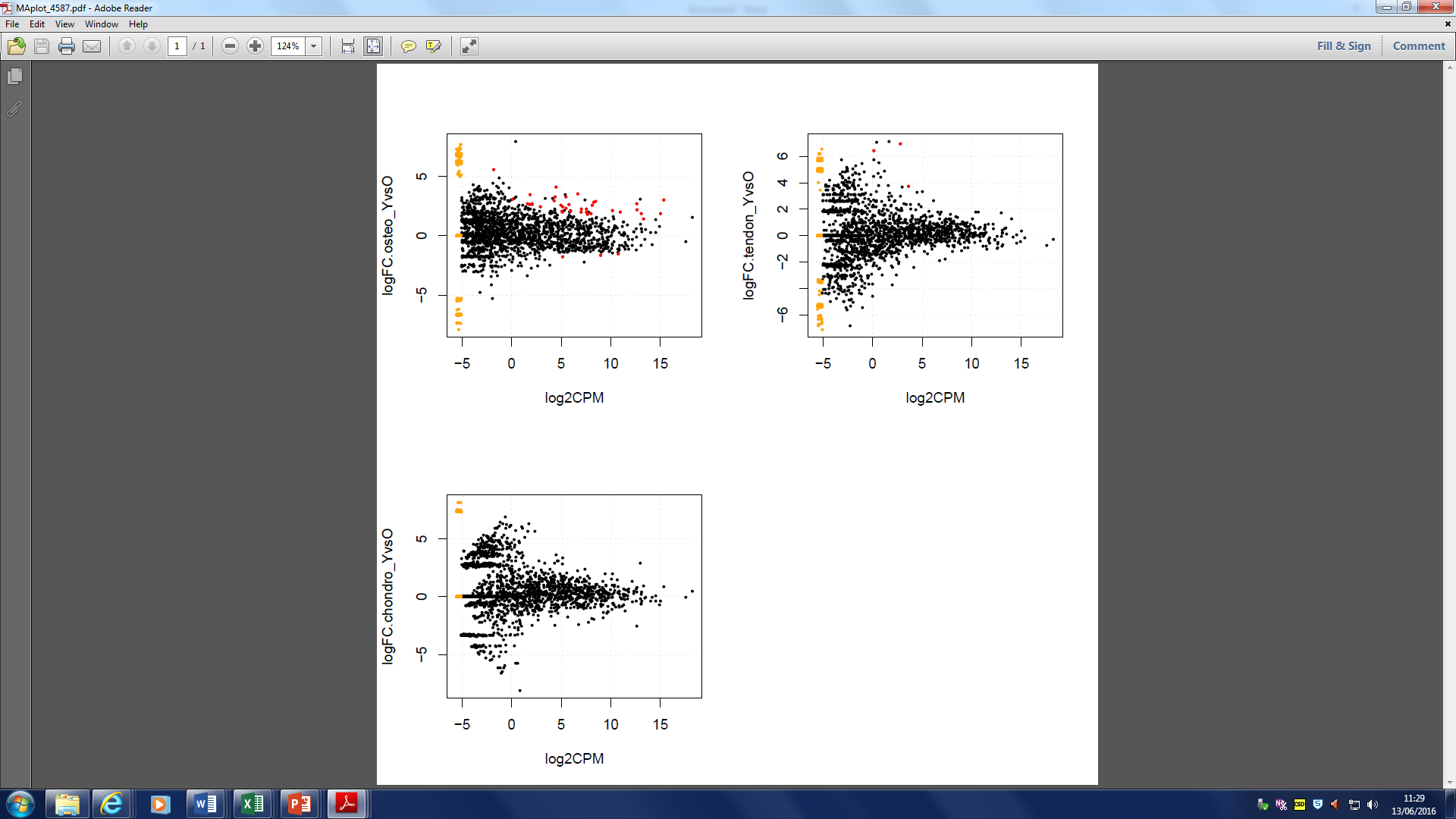

Supplement: S8 File — (DOCX) [file pone.0160517.s008.docx]
