# Supplementary material for: Decoding the Regulatory Landscape of Ageing in Musculoskeletal Engineered Tissues Using Genome-Wide DNA Methylation and RNASeq
Source: PLoS One. 2016 Aug 17;11(8):e0160517. doi: 10.1371/journal.pone.0160517 (PMC4988628; doi:10.1371/journal.pone.0160517)
Supplement: S10 File — (DOCX) [file pone.0160517.s010.docx]

Supplementary file 8- MicroRNA-mRNA expression pairing using DE miRs and mRNA from osteogenic and tenogenic engineered tissues using the filters adjusted P<0.05 and 1.4 log_2_ fold change. A positive miR fold change relates to reduced expression in constructs derived from old MSCs.

| **Construct Type** | **miR identification** | **miR Log_2_ Fold Change** | **Confidence** | **mRNA target identification** | **mRNA Log_2_ Fold Change** |
| --- | --- | --- | --- | --- | --- |
| Osteogenic | hsa-let-7i-5p | 1.9 | High | ADAMTS15 | -4.4 |
|  | hsa-let-7i-5p | 1.9 | High | ADAMTS5 | -3.0 |
|  | hsa-let-7i-5p | 1.9 | High | NOVA1 | -5.6 |
|  | hsa-let-7i-5p | 1.9 | High | SLC6A15 | -7.3 |
|  | hsa-miR-140-5p | 2 | High | ADAMTS5 | -3.0 |
|  | hsa-miR-140-5p | 2 | High | MMD | -2.4 |
|  | hsa-miR-143-3p | 3 | Moderate | IL18 | 3.4 |
|  | hsa-miR-143-3p | 3 | High | NOVA1 | -5.6 |
|  | hsa-miR-148a-3p | 2.7 | High | ADAMTS15 | -4.4 |
|  | hsa-miR-148a-3p | 2.7 | High | ADAMTS5 | -3.0 |
|  | hsa-miR-148a-3p | 2.7 | Moderate | DPP4 | -3.4 |
|  | hsa-miR-148a-3p | 2.7 | Moderate | IL1RL1 | 4.1 |
|  | hsa-miR-148a-3p | 2.7 | High | NOVA1 | -5.6 |
|  | hsa-miR-148a-3p | 2.7 | High | TGFA | 5.6 |
|  | hsa-miR-487a-3p | 2.7 | Moderate | CNTNAP4 | -5.2 |
|  | hsa-miR-487a-3p | 2.7 | Moderate | RAI2 | -4.5 |
|  | hsa-miR-487a-3p | 2.7 | Moderate | TMEM178A | 3.5 |
|  | hsa-miR-21-3p | 1.8 | Moderate | CNTNAP4 | -5.2 |
|  | hsa-miR-21-3p | 1.8 | Moderate | MAB21L1 | -3.4 |
|  | hsa-miR-21-3p | 1.8 | Moderate | TBX18 | -1.8 |
|  | hsa-miR-22-3p | 1.4 | High | ADAMTS5 | -3.0 |
|  | hsa-miR-22-3p | 1.4 | Moderate | DPP4 | -3.4 |
|  | hsa-miR-22-3p | 1.4 | High | MAPK10 | -2.1 |
|  | hsa-miR-22-3p | 1.4 | Moderate | NOVA1 | -5.6 |
|  | hsa-miR-22-3p | 1.4 | Moderate | NTNG1 | -2.5 |
|  | hsa-miR-27b-3p | 1.8 | High | ADAMTS5 | -3.0 |
|  | hsa-miR-27b-3p | 1.8 | High | FAM65B | -3.5 |
|  | hsa-miR-27b-3p | 1.8 | High | MAPK10 | -2.1 |
|  | hsa-miR-27b-3p | 1.8 | High | MMD | -2.4 |
|  | hsa-miR-27b-3p | 1.8 | High | NOVA1 | -5.6 |
|  | hsa-miR-30a-5p | 2.1 | High | ADAMTS5 | -3.0 |
|  | hsa-miR-30a-5p | 2.1 | High | CACHD1 | -1.8 |
|  | hsa-miR-30a-5p | 2.1 | Moderate | DSG2 | -5.0 |
|  | hsa-miR-30a-5p | 2.1 | High | MAB21L1 | -3.4 |
|  | hsa-miR-30a-5p | 2.1 | High | MMD | -2.4 |
|  | hsa-miR-30a-5p | 2.1 | High | NOVA1 | -5.6 |
|  | hsa-miR-30a-5p | 2.1 | High | NTNG1 | -2.5 |
|  | hsa-miR-30a-5p | 2.1 | High | SLC6A15 | -7.3 |
|  | hsa-miR-30a-5p | 2.1 | High | TNXB | -6.8 |
|  | hsa-miR-340-5p | 2.2 | High | ADAMTS5 | -3.0 |
|  | hsa-miR-340-5p | 2.2 | High | FAM65B | -3.5 |
|  | hsa-miR-340-5p | 2.2 | High | SULT1B1 | -5.5 |
|  | hsa-miR-369-3p | 3.5 | Moderate | MMD | -2.4 |
|  | hsa-miR-655-3p | 2.6 | Moderate | DPP4 | -3.4 |
|  | hsa-miR-655-3p | 2.6 | Moderate | TBX18 | -1.8 |
|  | hsa-miR-455-5p | 2.8 | Moderate | SLC6A15 | -7.3 |
|  | hsa-miR-455-5p | 2.8 | High | TBX18 | -1.8 |
|  | hsa-miR-493-3p | 2.1 | High | MAB21L2 | -7.0 |
|  | hsa-miR-495-3p | 2.6 | High | MAPK10 | -2.1 |
|  | hsa-miR-495-3p | 2.6 | High | NOVA1 | -5.6 |
|  | hsa-miR-495-3p | 2.6 | High | RAI2 | -4.5 |
|  | hsa-miR-495-3p | 2.6 | High | SLC6A15 | -7.3 |
|  | hsa-miR-495-3p | 2.6 | High | TMEFF2 | -3.4 |
|  | hsa-miR-542-3p | 3.3 | High | NOVA1 | -5.6 |
| Tenogenic | hsa-miR-548j-5p | 6.4 | Moderate | PPARG | -3.0 |
